# Metabolome and transcriptome analyses reveal metabolic differences and identify candidate regulatory factors among three habitat types of *Gentiana scabra* Bunge

**DOI:** 10.3389/fpls.2026.1761588

**Published:** 2026-04-29

**Authors:** Junnan Du, Jiayi Liu, Xuelin Yu, Jiayi Luo, Jie Sun, Dan Wang, Haibo Yin

**Affiliations:** School of Pharmacy, Liaoning University of Traditional Chinese Medicine, Dalian, Liaoning, China

**Keywords:** *Gentiana scabra* Bunge, habitat, iridoid compounds, metabolomics, transcriptomics

## Abstract

**Introduction:**

*Gentiana scabra* Bunge, a primary botanical source of the traditional Chinese medicine *Gentianae Radix* et *Rhizoma*. The quality of *G. scabra* varies under different habitats, but the underlying mechanism remains unclear.

**Methods:**

In this study, comparative analyses of UPLC-MS/MS-based metabolomics and RNA-seq-based transcriptomics were integrated to reveal differences in metabolite accumulation among *G. scabra* grown in three habitats: wild shrublands, wild meadows, and cultivated farmlands.

**Results:**

A total of 1824 metabolites and 93076 Unigenes were identified across *G. scabra* from the three habitats. Specifically, 309 differential metabolites (DMs) and 645 differentially expressed genes (DEGs) were detected between the wild meadow group (YC) and wild shrubland group (YG), while 348 DMs and 605 DEGs were observed between the cultivated farmland group (ZP) and wild meadow group (YC).A total of 510 DMs and 917 DEGs were identified between the cultivated farmland group (ZP) and wild shrubland group (YG), which reveals differences in metabolite accumulation of *G. scabra* among the three habitats. Integrated analysis further identified genes associated with the quality formation of *G. scabra* medicinal materials, and these genes play crucial roles in the accumulation of metabolic compounds in *G. scabra*. Genes such as 3-hydroxy-3-methylglutaryl-CoA reductase (*HMGR*), diphosphomevalonate decarboxylase (*MVD*), and isopentenyl diphosphate isomerase (*IDI*) may be involved in regulating terpenoid backbone biosynthesis.

**Discussion:**

DMs were mainly enriched in “metabolic pathways” and “environmental information processing”, which may be attributed to the effects of environmental factors in the growing habitats of *G. scabra*. The expression levels of four genes, namely *HSFF, SCPL-I, LOC107801770*, and *CYP71D55*, can serve as candidate genes for distinguishing *G. scabra* from the three habitats. This study provides a theoretical basis for future molecular breeding and “simulative habitat cultivation” of *G. scabra*.

## 1 Introduction

*Gentiana scabra* Bunge is a perennial herb belonging to the genus *Gentiana* (Gentianaceae). It is mainly distributed on sunny slopes, forest edges, grasslands, or river banks (Wu et al., 2013). As one of the original plant sources of the traditional Chinese medicine *Gentianae Radix* et *Rhizoma* recorded in the Pharmacopoeia of the People’ s Republic of China (National Pharmacopoeia Commission, 2025), it possesses the effects of clearing heat and drying dampness, as well as purging fire from the liver and gallbladder. Its main chemical constituents include iridoids, flavonoids, triterpenoids, xanthones, and amino acids (Fu et al., 2025). Modern pharmacological studies have demonstrated that it exhibits antibacterial, anti-inflammatory, hepatoprotective, antimalarial, sedative, antihypertensive, and stomachic activities (Liu and Zhang, 2025). *G. scabra* is a genuine medicinal herb in Northeast China and also one of the “Six Treasures of Liaoning Medicinal Materials”. It is widely used in traditional Chinese medicine prescriptions such as Longdan Xiegan Decoction (Hu et al., 2021; Pan et al., 2024), Liangjing Wan (Wang et al., 2023), and Danggui Longhui Wan (Li et al., 2024), with a large market demand.

At present, *G. scabra* in the market is mainly derived from wild and cultivated resources. Due to excessive excavation, wild resources have become extremely scarce. However, farmland-cultivated gentian suffers from problems such as uneven quality, mainly reflected in non-standard introduction and domestication of wild germplasm, as well as imperfect breeding and screening systems for improved varieties. With the increase of cultivation years, phenomena such as variety degeneration, yield reduction, and quality decline have gradually emerged (Wang et al., 2024). During short-term introduction, farmers often adopt inappropriate cultivation measures including large-scale continuous cropping, excessive application of chemical fertilizers and pesticides, and shortening of the cultivation cycle. These practices alter the physiological and biochemical processes of medicinal plants, further affecting the biosynthesis and accumulation of secondary metabolites, and ultimately exert impacts on the quality, safety, and therapeutic efficacy of medicinal materials (Lyu et al., 2025).

Environmental conditions are key factors determining the quality of medicinal plants. Most medicinal plants have formed specific adaptations to the microenvironment of their native habitats after long-term adaptation, and there are significant differences between native habitats and farmland cultivation environments (Lyu et al., 2025). Previous studies have shown that medicinal material quality is co-regulated by cultivation methods and environmental conditions, which further lead to differences in the biosynthesis and accumulation of secondary metabolites (Guo et al., 2020). *G. scabra* is mainly distributed in Liaoning, Jilin, Heilongjiang and other provinces in China (Ren et al., 2020), and its wild populations naturally occur in various habitats such as shrubs and grasslands. Our previous studies have found that the quality of *G. scabra* from different habitats varies. However, how habitat differences affect the accumulation of secondary metabolites at the molecular level and its underlying regulatory mechanism remain unclear. Therefore, it is urgent to screen suitable growth environments for maintaining the quality of *G. scabra* and to clarify the intrinsic regulatory mechanism by which habitat variation affects metabolite accumulation.

Based on field sampling and investigation, *G. scabra* in this study was classified into three groups: wild populations in shrub habitats (YG), wild populations in meadow habitats (YC), and farmland-cultivated populations (ZP). Using integrated transcriptomic and metabolomic analyses, the objectives were to: compare the differences in metabolites among the three populations;screen the significantly DMs and their related genes; and explore the potential regulatory pathways affected by habitat differences. This study can provide a scientific basis for the quality grading and production evaluation of *Gentianae Radix* et *Rhizoma*, and lay a foundation for the future molecular breeding of *G. scabra* as well as the strategy of “habitat-simulating cultivation”.

## 2 Materials and methods

### 2.1 Plant materials

Fresh roots and rhizomes of *G. scabra* were collected from three different ecological types across various producing areas in Heilongjiang, Jilin, and Liaoning Provinces (**Table 1**). Voucher specimens were deposited in the Herbarium of Chinese Medicinal Resources, Liaoning University of Traditional Chinese Medicine. After cleaning, the fresh root and rhizome tissues were cut into small pieces, immediately frozen in liquid nitrogen, and then stored at -80 °C for subsequent use. Among the selected sampling sites: the wild shrub habitat was well-drained sloped land accompanied by mixed shrub vegetation, forming a semi-shaded growing environment (canopy cover exceeding 30%); the wild meadow habitat was open grassland with sufficient light availability, corresponding to site conditions with relatively uniform interspecific competition among herbaceous plants; the artificial farmland habitat was leveled plots under standardized tillage, irrigation and fertilization management, representing a growing environment free from significant interspecific plant competition. Detailed environmental characteristics for each sampling site are provided in **Supplementary Table 11**.

**Table 1:** Summary of *Gentiana scabra* samples used in this study.

Sample Code	Sampling Location	Habitat Type	Group Code
A1-1	Dongshan District, Hegang City, Heilongjiang Province	Wild Shrubland	YG
A1-2	Liaoyuan City, Jilin Province	Wild Shrubland	YG
A1-3	Shangde Village, Xifeng Town, Xifeng County, Liaoning Province	Wild Shrubland	YG
B2-1	Zhuanghe City, Dalian City, Liaoning Province	Wild Meadow	YC
B2-2	Pulandian District, Dalian City, Liaoning Province	Wild Meadow	YC
B2-3	Yangmuchuan Town, Kuandian County, Dandong City, Liaoning Province	Wild Meadow	YC
C3-1	Yingemen Town, Qingyuan County, Fushun City, Liaoning Province	Cultivated Farmland	ZP
C3-2	Tukouzi Township, Qingyuan County, Fushun City, Liaoning Province	Cultivated Farmland	ZP
C3-3	Longquan Town, Jingyu County, Baishan City, Jilin Province	Cultivated Farmland	ZP

### 2.2 Sample preparation and extraction

The root tissue samples of *G. scabra* were vacuum freeze-dried in a freeze dryer (Scientz-100F), then ground into powder at 30 Hz for 1.5 minutes using a grinder (MM400, Retsch). A 50 mg aliquot of the sample powder was accurately weighed with an electronic balance (MS105DM) and extracted by adding 1200 μL of pre-cooled 70% methanol aqueous solution (-20 °C). Vortex mixing was performed 6 times, with each mixing lasting 30 seconds at 1-minute intervals. After centrifugation at 12000 rpm for 3 minutes, the supernatant was collected, filtered through a microfiltration membrane (0.22 μm pore size), and stored in an injection vial for subsequent UPLC-MS/MS analysis (Sun et al., 2019).

### 2.3 Chromatographic and mass spectrometric acquisition conditions

The data acquisition instrument system mainly included an Ultra Performance Liquid Chromatography (UPLC) system (ExionLCT™ AD, https://sciex.com.cn/) and a tandem mass spectrometry (MS/MS) system. The liquid chromatography conditions were as follows: Column: Agilent SB-C18 (1.8 μm, 2.1 mm × 100 mm); Mobile phase: Phase A was ultrapure water (containing 0.1% formic acid), and Phase B was acetonitrile (containing 0.1% formic acid); Elution gradient: The initial proportion of Phase B was 5% at 0.00 min, linearly increased to 95% within 9.00 min, maintained at 95% for 1 min, decreased to 5% from 10.00 to 11.10 min, and equilibrated at 5% until 14 min; Flow rate: 0.35 mL/min; Column temperature: 40 °C; Injection volume: 2 μL.

The mass spectrometry conditions were as follows: Ion source: Electrospray ionization (ESI) with a temperature of 500 °C; Ion spray voltage (IS): 5500 V (positive ion mode)/-4500V (negative ion mode); Ion source gases and curtain gas: Gas I (GSI), Gas II (GSII), and curtain gas (CUR) were set to 50, 60, and 25 psi, respectively; collision-induced dissociation (CID) parameter was set to high; QQQ scanning mode: Multiple reaction monitoring (MRM) mode was used, with collision gas (nitrogen) set to medium; Parameter optimization: Declustering potential (DP) and collision energy (CE) for each MRM ion pair were optimized individually; MRM ion pair monitoring: A specific set of MRM ion pairs was monitored during each period according to the metabolites eluted in the corresponding time window (Zhang et al., 2022; Lin et al., 2024).

### 2.4 Qualitative and quantitative analysis of metabolites

Qualitative and quantitative analysis of metabolites was performed on a UPLC-MS/MS platform via the multiple reaction monitoring (MRM) mode of triple quadrupole mass spectrometry. Samples were injected in a random sequence, and quality control (QC) samples—prepared by mixing the extracts of all samples—were inserted at an interval of one QC sample per six experimental samples to monitor the stability of the analytical system. Raw data were acquired using Analyst 1.6.3, and qualitative identification was performed by matching the retention time, characteristic ion pairs and collision energy against an in-house database (MWDB). Isotopic peaks, redundant peaks formed by the adduction of K^+^, Na^+^ or NH_4_^+^, and signals identified as fragment ions of other metabolites with higher molecular weights were excluded from the dataset. Peak integration was performed using MultiQuant 3.0.2, and the relative content was represented by peak area. After the data were processed with median normalization, only metabolites with a coefficient of variation (CV) below 30% in quality control (QC) samples were retained for subsequent analyses. The screening of DMs combined multivariate and univariate statistics: based on the orthogonal partial least squares-discriminant analysis (OPLS-DA) model validated by permutation test, metabolites with a variable importance in projection (VIP) value greater than 1 were selected, followed by two-tailed t-test (adjusted by false discovery rate, FDR) and fold change analysis. Ultimately, metabolites that simultaneously met the thresholds of VIP ≥ 1, FDR ≤ 0.05 and fold change ≥ 2 or fold change ≤ 0.5 were defined as significantly DMs. To evaluate the impact of multiple testing correction, we compared the number of DMs before and after FDR adjustment (**Supplementary Table 9**).

### 2.5 RNA extraction, sequencing, and *De Novo* assembly

Transcriptome sequencing was performed on *G. scabra* roots from three different habitat types, with three biological replicates set for each type. The RNA-seq analysis was conducted by MetWare Biotechnology Co., Ltd. (Wuhan, China). Total RNA was extracted using the CTAB method (Johnson et al., 2014; Pan et al., 2023). The integrity of sample RNA and the presence of DNA contamination were analyzed by agarose gel electrophoresis. RNA quality was evaluated with a NanoPhotometer spectrophotometer to determine RNA purity (OD260/280 and OD260/230 ratios). The RNA concentration was measured using a Qubit 2.0 Fluorometer. The RNA integrity number (RIN) was then assessed with an Agilent 2100 Bioanalyzer. Library construction was performed using Illumina’s NEBNext^®^ Ultra™ RNA Library Prep Kit. After library construction, initial quantification was conducted with a Qubit 2.0 Fluorometer, and the libraries were diluted to 1.5 ng/μL. Subsequently, the insert size of the libraries was detected using an Agilent 2100 Bioanalyzer. Once the insert size met the expected requirements, the effective concentration of the libraries was accurately quantified by qRT-PCR (effective concentration > 2 nM) to ensure library quality. After passing library quality inspection, different libraries were pooled in proportion and subjected to 150 bp paired-end (PE150) sequencing on the *Illumina NovaSeq 6000 platform* (Grabherr et al., 2011; Wang et al., 2021).

Clean Reads were obtained from the raw data following quality control with fastp. All sample Clean Reads were subjected to *de novo* mixed assembly using Trinity (v2.13.2), and redundancy was removed via Corset clustering, ultimately yielding 93,076 non-redundant Unigenes as the reference transcriptome. To systematically evaluate the quality and reliability of the *de novo* assembly, a multi-dimensional analysis was performed: the Unigenes had an N50 of 2,076 bp and an average length of 1,414 bp, indicating good assembly continuity (**Supplementary Table 1**). BUSCO assessment based on the Viridiplantae_odb10 dataset showed that the vast majority of conserved single-copy orthologs were fully assembled (completeness ≥90%), confirming the biological integrity of the assembly (**Supplementary Figure 1**). To directly verify the representativeness of the assembly relative to the raw data, all sample Clean Reads were mapped back to the Unigene reference sequences using Bowtie2 (via the RSEM pipeline), resulting in an average total mapping rate of 85.9% (84.6-87.2%) (**Supplementary Table 2**). This demonstrates that the reference transcriptome is reliable and suitable for subsequent expression quantification and differential analysis.

### 2.6 Validation of RNA-seq data by qRT-PCR

First-strand cDNA was synthesized from total RNA using the FastKing cDNA First-Strand Synthesis Kit (gDNA removal) (Tiangen, KR116) for qRT-PCR validation. QRT-PCR was carried out on an ABI 7500 Real-Time PCR System, with reactions performed using SYBR^®^ Premix Ex Taq™ II (Tli RNase H Plus) and ROX plus (RR82LR) according to the manufacturer’s instructions (Takara Bio, Kusatsu, Japan). Primers were synthesized by Sangon Biotech (Shanghai) Co., Ltd. β-actin was used as the reference gene, and key DEGs in the compound synthesis pathways were selected to verify their expression levels via quantitative reverse transcription-polymerase chain reaction (qRT-PCR).

The qRT-PCR reaction system (10 μL total volume) consisted of 5 μL of 2×SYBR Green PCR Master Mix, 0.05 μL of QN ROX Reference Dye, 0.7 μL each of forward and reverse primers, 1 μL of cDNA template, and 2.55 μL of ddH_2_O. The reaction program was set as follows: pre-denaturation at 95 °C for 2 min; 40 cycles of amplification (95 °C for 5 s, 60 °C for 30 s); and a melting curve stage where the temperature was increased from 50 °C to 95 °C at a rate of 0.5 °C every 5 s. Three technical replicates were set for each sample to obtain Ct values, and the relative expression levels were calculated using the 2^-ΔΔCt^ method (Livak and Schmittgen, 2001). Statistical significance was determined by one-way ANOVA followed by Tukey’s honestly significant difference (HSD) *post-hoc* test using SPSS 26.0. Data are presented as mean ± standard deviation (SD) of three biological replicates. The primer sequences used in the qPCR analysis are listed in **Supplementary Table 3**.

### 2.7 Gene annotation and differential gene expression analysis

To obtain reference sequences for transcriptome analysis, clean reads from all samples were pooled and assembled using Trinity (version 2.8.5) with default parameters (Grabherr et al., 2011). The resulting unigenes were used as the reference sequences. Functional annotation of these unigenes was performed by aligning them against public databases, including Non-Redundant Protein (NR), Non-Redundant Nucleotide (NT), Pfam, KOG/COG, SWISS-PROT, KEGG Orthology (KO), and Gene Ontology (GO) databases. Sequence reads from each sample were then remapped to the reference unigenes using RSEM software (Li and Dewey, 2011). Gene expression levels were measured as FPKM (Fragments Per Kilobase of transcript per Million mapped fragments) based on uniquely mapped reads. For genes with more than one alternative transcript, the longest transcript was selected for FPKM calculation. Differential expression analysis among the three groups was conducted using the DESeq R package (version 1.10.1). DESeq provides statistical routines to identify DEGs in digital gene expression data using a model based on the negative binomial distribution. The Benjamini and Hochberg method was used to control the false discovery rate and adjust the resulting P-values. Genes with an adjusted P-value < 0.05 identified by DESeq were considered differentially expressed. The Audic method (Audic and Claverie, 1997) was applied to screen for differences in gene expression in *G. scabra* roots among the three habitats. To evaluate the impact of multiple testing correction, we compared the number of DEGs before and after FDR adjustment.(**Supplementary Table 10**).

### 2.8 Correlation analysis of metabolomic and transcriptomic data

Pearson correlation coefficients were calculated for the integration of metabolomic and transcriptomic data. Prior to analysis, log_2_ transformation was uniformly performed on all data. For the integrated analysis of metabolomic and transcriptomic data, a threshold of Pearson correlation coefficient > 0.8 was set to screen for correlations between key metabolites and DEGs. The relationships between metabolomic and transcriptomic data were visualized using Cytoscape software.

## 3 Results

### 3.1 Comparison of metabolites in *G. scabra* from three habitats

Positive and negative ions were obtained from the total ion chromatograms (TICs) of UPLC-MS/MS. A total of 1824 metabolites were quantified, including 440 primary metabolites and 1384 secondary metabolites. The most abundant metabolites among the three ecotypes were terpenoids, phenolic acids, amino acids and their derivatives, alkaloids, flavonoids, lipids, lignans and coumarins, organic acids, nucleotides and their derivatives, and quinones. DMs were mainly found in terpenoids (17.93%), followed by phenolic acids (13.98%), amino acids and their derivatives (13.93%), and alkaloids (9.76%) (**Supplementary Figure 2**).

**Figure 1:**
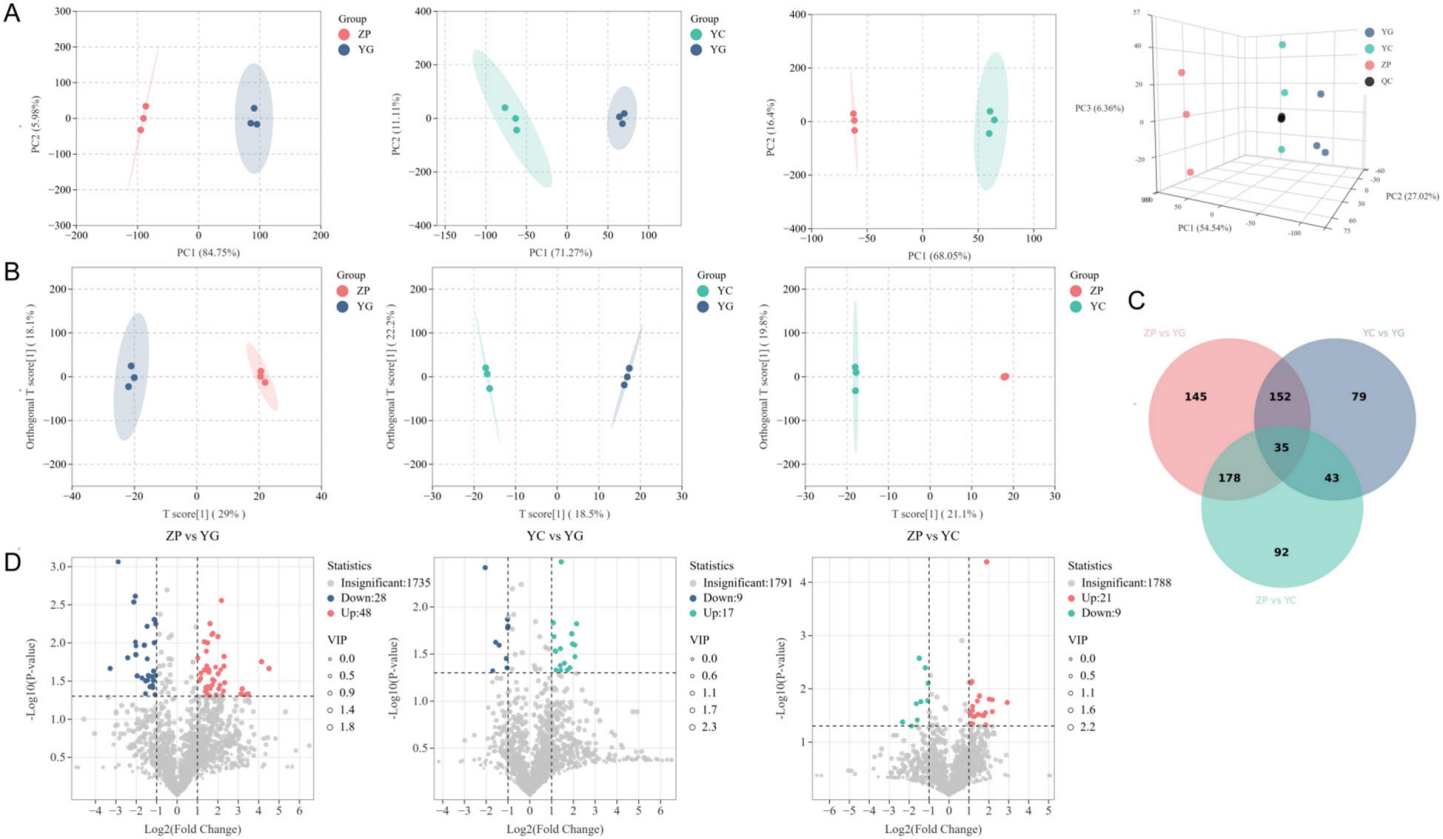
Metabolite distribution of *G. scabra*. from three habitats. **(A)** Principal component analysis (PCA) score plot of metabolite profiles. Each dot represents one biological replicate (n=3 per group). PC1, PC2, and PC3 explain 54.54%, 27.02%, and 6.36% of total variance, respectively. **(B)** Orthogonal partial least squares-discriminant analysis (OPLS-DA) score plot with 200 permutation test validation. Q² and P-values for each comparison are indicated. **(C)** Venn diagram showing unique and shared differential metabolites (DMs) among three pairwise comparisons. **(D)** Volcano plots displaying -log^10^(P-value) versus log^2^(fold change) for DMs. Red dots: significantly upregulated; blue dots: significantly downregulated; gray dots: non-significant. VIP ≥ 1, FDR < 0.05, |fold change| ≥ 2. YG: Wild shrubby *G. scabra*; YC: Wild meadow *G. scabra*; ZP: Cultivated farmland *G. scabra*.

Principal component analysis (PCA) results (**Figure 1A**) showed that biological replicates of *G. scabra* from the three habitats clustered in distinct regions. The first principal component explained 54.54% of the total variability, the second principal component explained 27.02%, and the third principal component explained 6.36%, indicating significant metabolic differences among them. Samples of wild shrubby *G. scabra* were mainly clustered on the right side, indicating that PC1 had a major impact on sample separation. Samples of wild meadow *G. scabra* were primarily concentrated in the middle, suggesting that PC3 exerted a significant effect on their separation. Cultivated farmland *G. scabra* samples were mostly grouped on the left side, indicating that PC2 made a certain contribution to their separation. Notably, cultivated farmland samples were far apart from the other two groups.

Under the OPLS-DA model (**Figure 1B**), permutation tests (200 permutations) showed: ZP vs. YG group (Q^2^ = 0.669, P = 0.05); ZP vs. YC group (Q^2^ = 0.504, P = 0.05); YC vs. YG group (Q^2^ = 0.582, P = 0.05). These P-values indicate that all three models were significant (P < 0.05) and free of overfitting. Since the Q^2^ values were >0.5, the models were considered stable and reliable.

The screening results were verified using a Venn diagram (**Figure 1C**) and volcano plots (**Figure 1D**). A total of 309 DMs were identified between the wild meadow group and wild shrubby group (237 upregulated, 72 downregulated). The cultivated farmland group and wild meadow group showed 348 DMs (207 upregulated, 141 downregulated). Additionally, 510 DMs were detected between the cultivated farmland group and wild shrubby group (367 upregulated, 143 downregulated). These DMs were mapped to the KEGG database for enrichment analysis among the three habitats (**Supplementary Figure 3**). The results indicated that the DMs were mainly enriched in “metabolic pathways”, “genetic information processing”, and “environmental information processing”.

**Figure 2:**
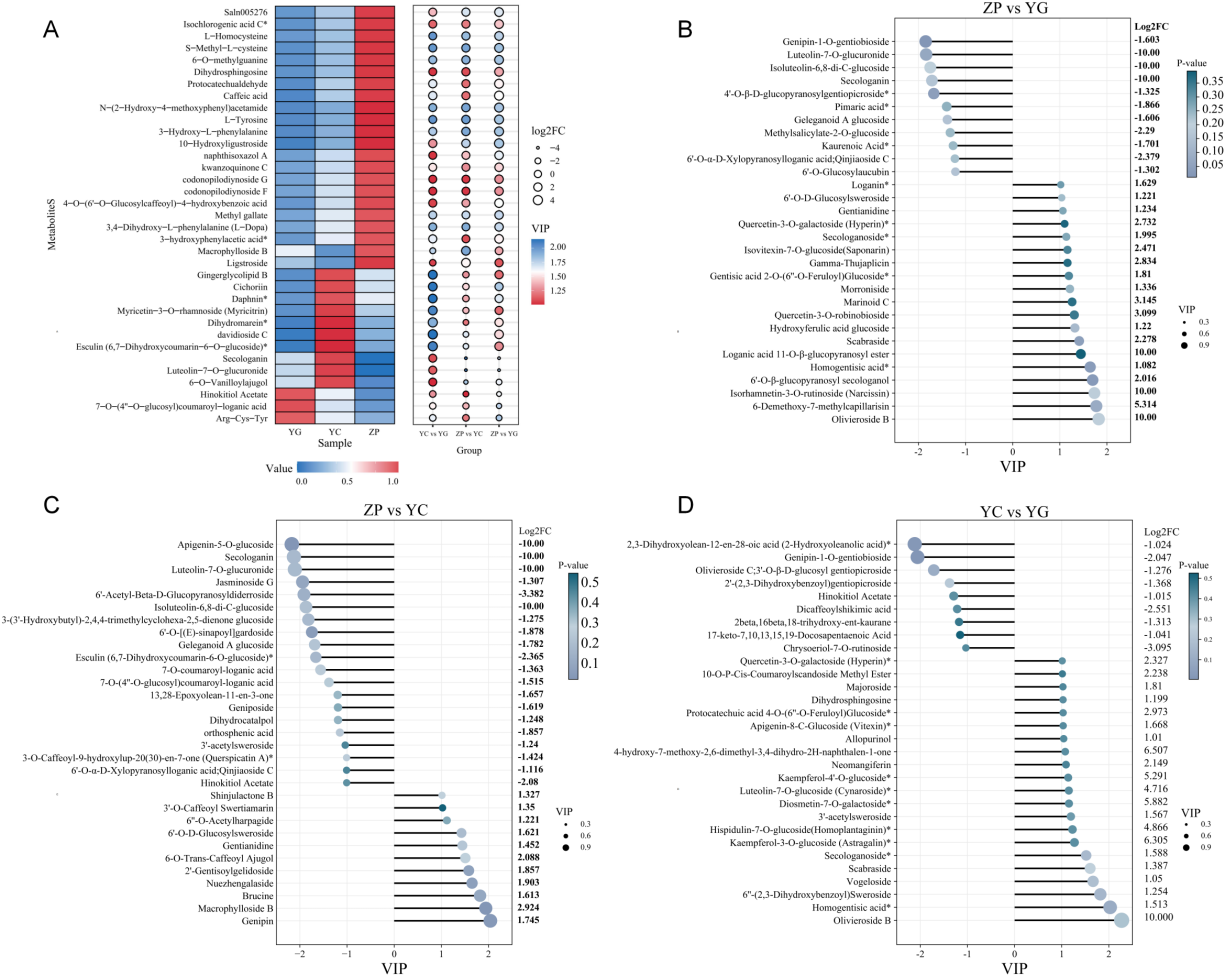
Accumulation patterns of differentially accumulated metabolites (DMs) in *Gentiana scabra* across three habitats. **(A)** Heatmap of 35 common DMs shared among the three habitat comparison groups. Red indicates high relative abundance, and blue indicates low relative abundance. **(B)** Lollipop plot showing DM differences between ZP and YG groups. **(C)** Lollipop plot showing DM differences between ZP and YC groups. **(D)** Lollipop plot showing DM differences between YC and YG groups. YG: Wild shrubby *G. scabra*; YC: Wild meadow *G. scabra*; ZP: Cultivated farmland *G. scabra*. VIP: variable importance in projection (a value > 1 indicates a significant contribution to group separation); Color scale indicates normalized metabolite abundance (z-score).

Thirty-five common DMs were identified from the intersection of the Venn diagram results among the three habitat comparison groups. Heatmap analysis of these 35 DMs (**Figure 2A**) revealed distinct differences in metabolite contents of *G. scabra* across different habitats. Among these, 22 DMs were highly abundant in the ZP group, dominated by amino acids and their derivatives as well as phenolic acids; 10 DMs were highly abundant in the YC group, primarily including lignins and coumarins; and 3 DMs were highly abundant in the YG group (**Supplementary Table 4**).

Key DMs related to the quality formation of *G. scabra* were screened. The contents of these key metabolites showed upward or downward trends across different habitats, with details as follows: ZP vs YG group (**Figure 2B**): Eleven metabolites, including genipin-1-O-gentiobioside, isovitexin-7-O-glucoside (Saponarin), isoluteolin-6,8-di-C-glucoside, secologanin, 4’-O-β-D-glucopyranosylgentiopicroside, and pimaric acid, had lower expression levels in the cultivated farmland habitat than in the wild shrubby habitat. Among them, 4’-O-β-D-glucopyranosylgentiopicroside, pimaric acid, and kaurenoic acid showed significant differences, and all three metabolites belonged to terpenoids. ZP vs YC group (**Figure 2C**): Twenty metabolites, such as apigenin-5-O-glucoside, secologanin, luteolin-7-O-glucuronide, and jasminoside G, exhibited lower expression levels in the cultivated farmland habitat compared to the wild meadow habitat. Significant differences were observed in esculin and 3-O-caffeoyl-9-hydroxylup-20(30)-en-7-one (querspicatin A). YC vs YG group (**Figure 2D**): Twenty-one metabolites, including olivieroside B, homogentisic acid, 6’’-(2,3-dihydroxybenzoyl) sweroside, vogeloside, scabraside, and secologanoside, had higher expression levels in the wild shrubby habitat than in the wild meadow habitat. Among these, homogentisic acid, secologanoside, kaempferol-4’-O-glucoside, protocatechuic acid 4-O-(6’’-O-Feruloyl) glucoside, apigenin-5-O-glucoside (cynaroside), kaempferol-3-O-glucoside (astragalin), diosmetin-7-O-galactoside, apigenin-8-C-glucoside (vitexin), and quercetin-3-O-galactoside (hyperin) showed significant differences.

### 3.2 DEGs of *G. scabra* in three habitats

Using RNA-seq technology, 6.67 Gb, 7.44 Gb, and 7.43 Gb of raw data were obtained from the three ecotype sample groups, with clean reads of 44,465,360, 49,633,843, and 49,553,363, respectively (**Supplementary Table 5**). After assembly, a total of 93,076 Unigenes were generated, with an average sequence length of 1,414 bp. Among these, 73.64% of the Unigenes were mainly distributed in the length range of 300–2000 bp, while 23.74% had lengths greater than 2000 bp. The Unigene sequences were aligned against the KEGG, NR, Swiss-Prot, GO, COG/KOG, and TrEMBL databases using DIAMOND BLASTX software to predict their potential functions. A total of 62,131 Unigenes (66.75%) were annotated in at least one database, while 33.25% of the Unigenes had no annotations in any database. Specifically, the numbers of annotations in the KEGG, NR, Swiss-Prot, TrEMBL, COG/KOG, and GO databases were 47,143, 60,229, 45,653, 59,798, 37,797, and 52,680, respectively (**Supplementary Table 6**).

**Figure 3:**
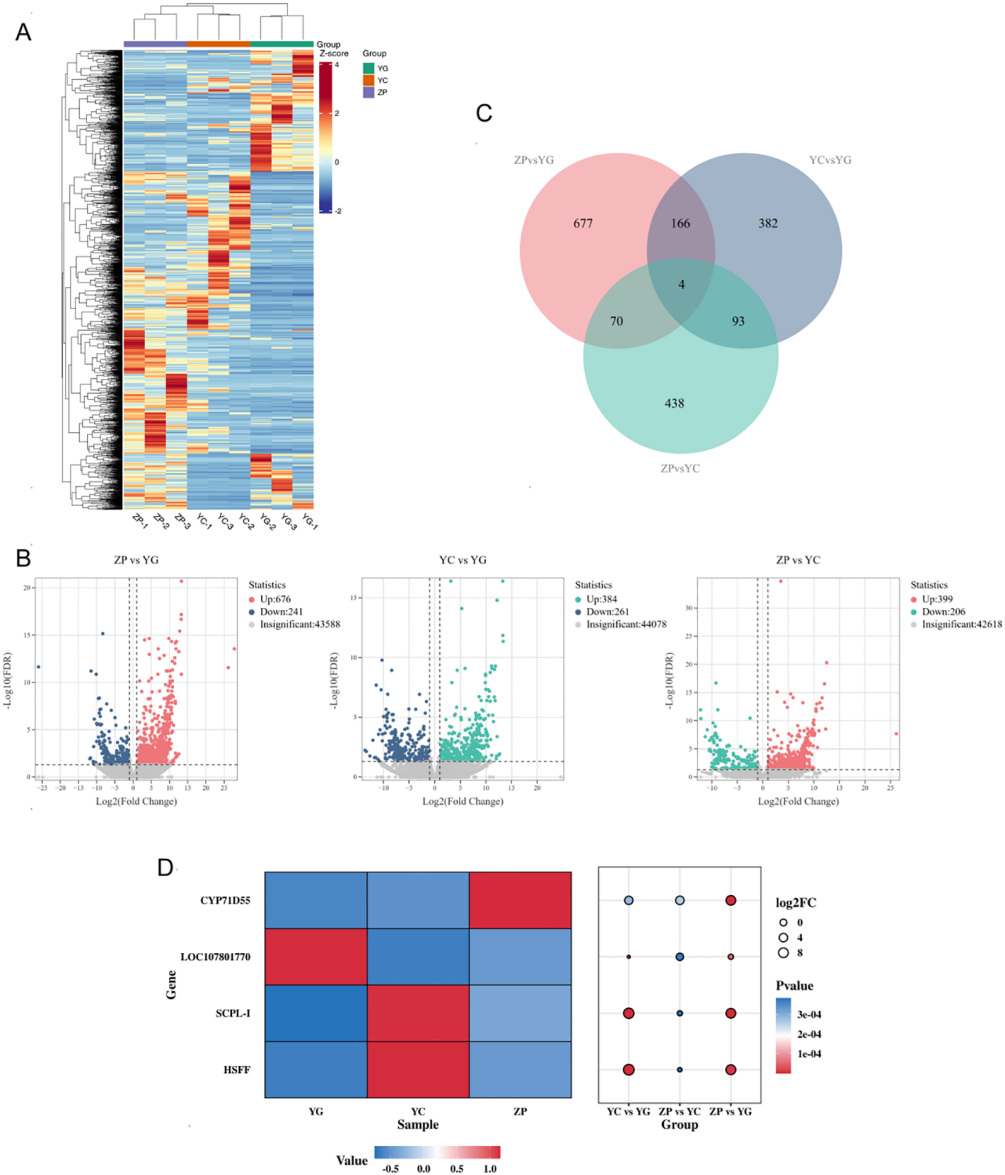
Transcriptional Profiles of *G. scabra*. Roots from Three Habitats **(A)** Cluster heatmap of DEGs; **(B)** Volcano plot showing log10 (P-value) and log_2_ (Fold Change) of DEGs; **(C)** Venn diagram of DEGs among different groups; **(D)** Bubble plot of DEGs. YG: Wild shrubby *G. scabra*; YC: Wild meadow *G. scabra*; ZP: Cultivated farmland *G. scabra*.

Transcriptome analysis results indicated that the gene expression levels of *G. scabra* varied among the three habitats. We found that DEGs were significantly enriched in KEGG pathways related to metabolism. The results of the DEG clustering heatmap (**Figure 3A**) showed that cultivated farmland *G. scabra* (ZP) was more similar to wild meadow *G. scabra* (YC), while there were obvious differences in the expression heatmap compared with wild shrubby *G. scabra* (YG). DEG statistics revealed that the number of DEGs between cultivated farmland (ZP) and wild shrubby (YG) *G. scabra* was the highest (917), including 676 upregulated and 241 downregulated genes. Meanwhile, 645 DEGs were identified between wild meadow (YC) and wild shrubby (YG) *G. scabra* (384 upregulated, 261 downregulated), and 605 DEGs between cultivated farmland (ZP) and wild meadow (YC) *G. scabra* (399 upregulated, 206 downregulated) (**Figure 3B**). Notably, four DEGs (*HSFF, SCPL-I, LOC107801770, and CYP71D55*) showed differential expression across all three habitats (**Figure 3C**), Bubble heatmap analysis was performed to investigate the expression patterns and significance of differential expression for four key DEGs (*HSFF, SCPL-I, LOC107801770, CYP71D55*) across three habitats (YG/YC/ZP) (**Figure 3D**). As shown in the left heatmap, these four genes exhibited distinct habitat-specific expression characteristics: *CYP71D55* (Cytochrome P450 71D55) was significantly up-regulated (red) in artificial farmland (ZP) but significantly down-regulated (blue) in wild shrubland (YG) and wild meadow (YC); *LOC107801770* (Locus 107801770, a putative oxidoreductase) was specifically up-regulated in wild shrubland (YG) with down-regulation in the other two habitats; *SCPL-I* (Serine Carboxypeptidase-Like acyltransferase I) and *HSFF* (Heat Shock Transcription Factor F) were only significantly up-regulated in wild meadow (YC) and down-regulated in the remaining habitats. The right bubble plot further validated the statistical significance of these differences: *CYP71D55* showed the largest and reddest bubble in the ZP vs. YG comparison, indicating the most significant expression difference (the lowest FDR-adjusted P-value and the highest log_2_FC) between artificial farmland and wild shrubland; *LOC107801770, SCPL-I* and *HSFF* all exhibited significant differences in the YC vs. YG comparison, among which *HSFF* had the largest bubble, suggesting that it is a core marker gene for distinguishing wild meadow from wild shrubland. These results indicated that the four genes may serve as key DEGs for distinguishing the habitats of *G. scabra.*

### 3.3 Functional classification of DEGs

**Figure 4:**
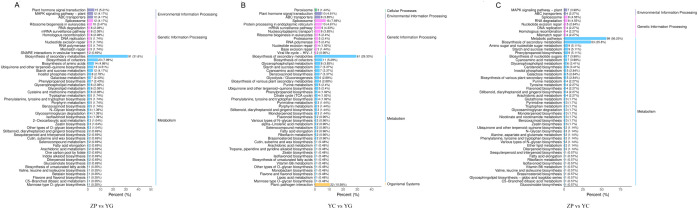
KEGG enrichment pathways of DEGs in *G. scabra* from three habitats. **(A)** KEGG enrichment pathways of DEGs between ZP and YG; **(B)** KEGG enrichment pathways of DEGs between YC and YG; **(C)** KEGG enrichment pathways of DEGs between ZP and YC. YG: Wild shrubby *G. scabra*; YC: Wild meadow *G. scabra*; ZP: Cultivated farmland *G. scabra*.

In KEGG enrichment classification, DEGs between cultivated farmland and wild shrubby *G. scabra*, they were enriched in 109 metabolic pathways (**Figure 4A**), including “metabolic pathways”, “genetic information processing”, and “environmental information processing”. DEGs between wild meadow and wild shrubby *G. scabra* were enriched in 99 metabolic pathways (**Figure 4B**), including “cellular processes”, “metabolic pathways”, “genetic information processing”, “environmental information processing”, and “organismal systems”. DEGs between cultivated farmland and wild meadow *G. scabra* were enriched in 92 metabolic pathways (**Figure 4C**), such as “metabolic pathways”, “genetic information processing”, and “environmental information processing”. Among these pathways, “metabolic pathways” and “biosynthesis of secondary metabolites” were the most abundant, which was consistent with the enrichment results of DMs. This indicated that there were significant differences in the secondary metabolite biosynthesis pathways of *G. scabra* across the three habitats.

**Figure 5:**
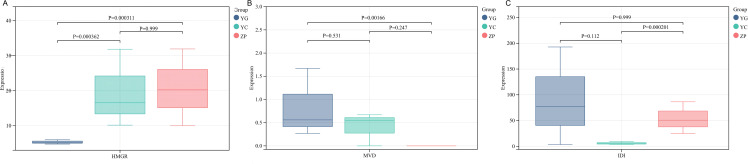
Boxplots showing the relative expression levels of three key genes in the terpenoid backbone biosynthesis pathway in Gentiana scabra across three habitats. **(A)** Relative expression level of HMGR gene; **(B)** Relative expression level of MVD gene; **(C)** Relative expression level of IDI gene. YG: Wild shrubby *G. scabra*; YC: Wild meadow *G. scabra*; ZP: Cultivated farmland *G. scabra*. Statistical significance was determined by one-way ANOVA, with P-values indicated above the plots.

Analysis of the KEGG pathways enriched by DEGs from the three comparison groups revealed 27 common pathways shared among the three contrasts (ZP vs. YG, ZP vs. YC, and YC vs. YG) (**Supplementary Table 7**). These common pathways included those related to the biosynthesis of bioactive components in *G. scabra*, such as “terpenoid backbone biosynthesis”, “sesquiterpenoid and triterpenoid biosynthesis”, and “plant-pathogen interaction”. Notably, *HMGR* (**Figure 5A**)*, MVD* (**Figure 5B**) and *IDI* (**Figure 5C**) in the “terpenoid backbone biosynthesis” pathway showed significant differences, which directly affect the biosynthesis of iridoid components.

To identify candidate genes involved in iridoid biosynthesis, the assembled unigenes were annotated by BLASTX (version 2.10.1) against the Kyoto Encyclopedia of Genes and Genomes (KEGG) database (http://www.kegg.jp/kegg/kegg1.html) with an E-value threshold of 10^-5^. Genes assigned to KEGG orthology terms associated with terpenoid backbone biosynthesis (map00900), monoterpenoid biosynthesis (map00902), and iridoid biosynthesis (map00903) were extracted. Additionally, sequences were compared against reference genes from characterized iridoid pathways in related species (e.g., Gentiana, Catharanthus roseus) using BLASTN and BLASTP to confirm homology. Only genes with significant alignment (E-value < 10^-10^) and conserved functional domains were retained as candidate genes. This approach yielded 25 candidate genes, including acetyl-CoA acetyltransferase (*ACAT*), hydroxymethylglutaryl-CoA synthase (*HMGCS*), hydroxymethylglutaryl-CoA reductase (*HMGR*), mevalonate kinase (*MVK*), phosphomevalonate kinase (*mvaK2*), diphosphomevalonate decarboxylase (*MVD*), isopentenyl phosphate kinase (*ipk*), 1-deoxy-D-xylulose-5-phosphate synthase (*dxs*), 1-deoxy-D-xylulose-5-phosphate reductoisomerase (*dxr*), 2-C-methyl-D-erythritol 4-phosphate cytidylyltransferase (*ispD*), 4-(cytidine 5’-diphospho)-2-C-methyl-D-erythritol kinase (*ispE*), 2-C-methyl-D-erythritol 2,4-cyclodiphosphate synthase (*ispF*), (E)-4-hydroxy-3-methylbut-2-en-1-yl diphosphate synthase (*gcpE*), (E)-4-hydroxy-3-methylbut-2-en-1-yl diphosphate reductase (*ispH*), isopentenyl-diphosphate delta-isomerase (*IDI*), isoprene synthase (*ispS*), farnesyl diphosphate synthase (*FDPS*), folate kinase (*FOLK*), isoprenylcysteine carboxyl methyltransferase (*ICMT*), Ste24p endopeptidase (*STE24*), precursor processing protease (*RCE1*), protein farnesyltransferase subunit beta (*FNTB*), dehydrodolichyl diphosphate synthase (*DHDDS*), chlorophyll synthase (*chlP*), and sucrose phosphate synthase (*SPS*). According to the gene expression data of *G. scabra* across the three habitats, three candidate genes (*HMGR, MVD, and IDI*) showed significant differences among groups. In the ZP vs YG group, both *HMGR* and *MVD* exhibited significant differences. *HMGR* had a higher expression level in the cultivated farmland habitat than in the wild shrubby habitat, while *MVD* showed higher expression in the wild shrubby habitat compared to the cultivated farmland habitat. In the ZP vs YC group, *IDI* displayed a significant difference, with higher expression in the cultivated farmland habitat than in the wild meadow habitat. In the YC vs YG group, *HMGR* showed a significant difference, with its expression level being the highest in the wild meadow habitat. To assess the robustness of pathway enrichment to FDR correction, we compared KEGG enrichment results using raw P-values vs. FDR-adjusted P-values. The core pathways related to terpenoid biosynthesis and secondary metabolism remained significant after FDR correction, while pathways with marginal significance were filtered out. This confirms the reliability of our main conclusions.

### 3.4 qRT-PCR validation of RNA-seq data

**Figure 6:**
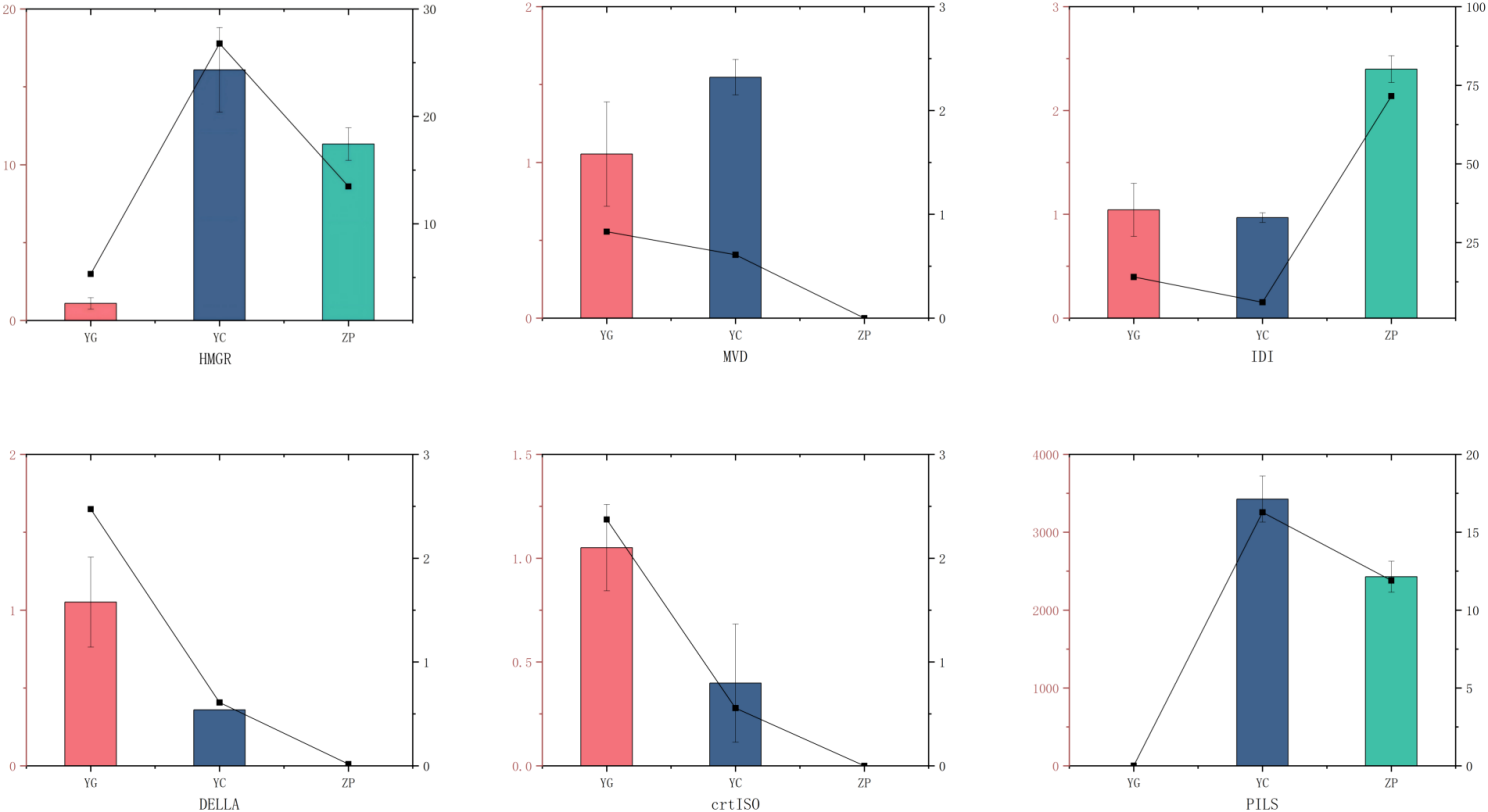
Quantitative real-time PCR (qRT-PCR) validation of six key differentially expressed genes (DEGs) in *G. scabra* across three habitats. The bar graphs represent the FPKM values from RNA-seq (left y-axis), and the line graphs represent the relative expression levels from qRT-PCR (right y-axis). Values are presented as mean ± standard deviation (SD) of three biological replicates. Statistical significance was determined by one-way ANOVA followed by Tukey’s honestly significant difference (HSD) *post-hoc* test (P < 0.05).

To validate the key RNA-seq results, six DEGs, namely *HMGR, MVD, IDI, DELLA, crtISO*, and *PILS* (**Figure 6**), were selected, and their expression levels across the three habitats were analyzed using qPCR. The expression patterns of these genes were consistent with the RNA-seq results, confirming the reliability of the RNA-seq data. Additionally, qPCR showed good consistency in the expression trends of both upregulated and downregulated genes.

### 3.5 Integrated analysis of transcriptome and metabolome

**Figure 7:**
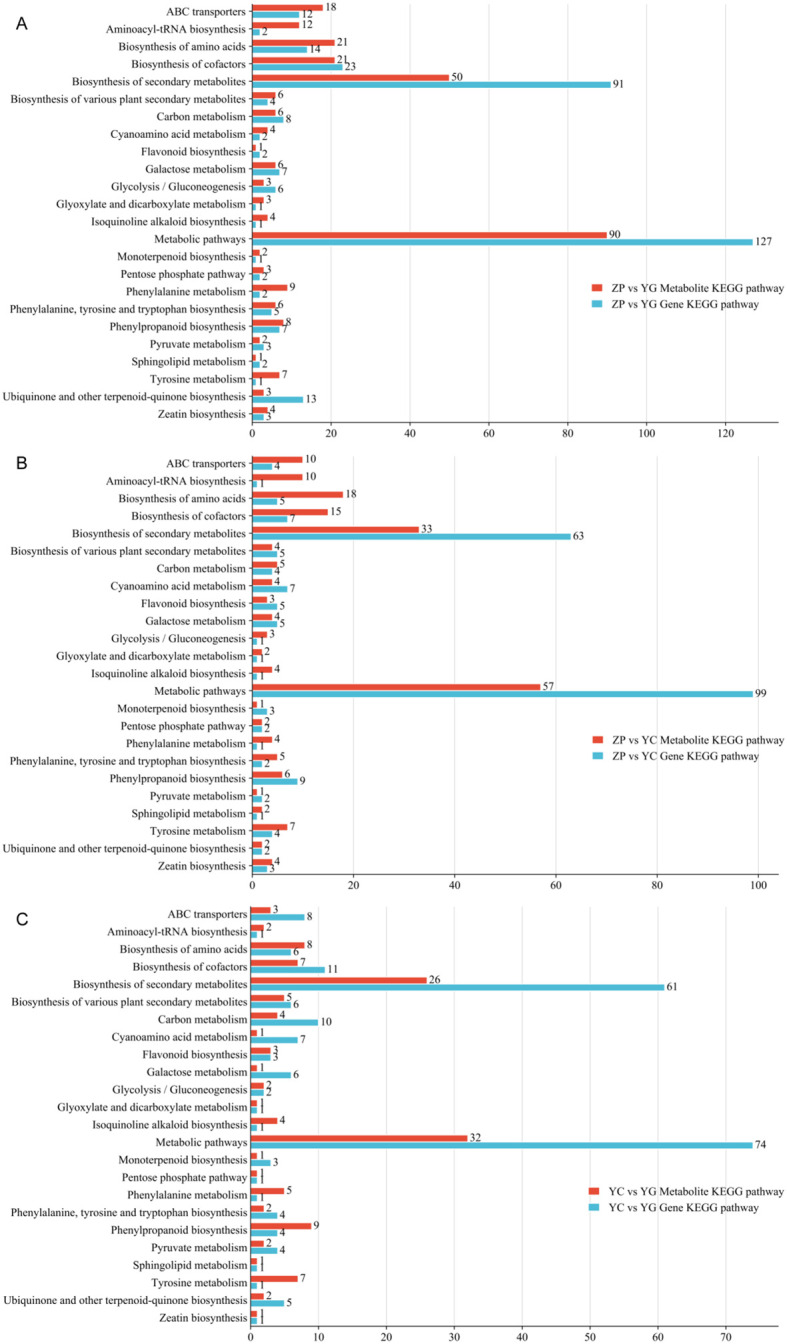
Integrated analysis of transcriptome and metabolome based on KEGG pathways in *G. scabra* across three comparison groups. **(A)** KEGG pathway enrichment for the ZP vs. YG comparison group; **(B)** KEGG pathway enrichment for the ZP vs. YC comparison group; **(C)** KEGG pathway enrichment for the YC vs. YG comparison group. Red bars represent the number of DMs enriched in each pathway, and blue bars represent the number of DEGs enriched in each pathway. All enrichment analyses were corrected for multiple testing using the false discovery rate (FDR) method, with a significance threshold of FDR < 0.05. YG: Wild shrubby *G. scabra*; YC: Wild meadow *G. scabra*; ZP: Cultivated farmland *G. scabra*. Three comparison groups were selected: ZP vs. YG, ZP vs. YC, and YC vs. YG. Specific names of KEGG pathways are provided on the left side of the bar chart, while the numbers of DEGs and DMs are shown on the right side.

To further clarify the relationship between metabolite accumulation and gene expression in the roots of *G. scabra* from different habitats, integrated biological annotation of the transcriptome and metabolome was performed. By combining metabolomic and transcriptomic data, we identified differential genes and DMs in the roots of *G. scabra* across different habitats. The KEGG pathways of DMs and differential genes from the three comparison groups (ZP vs. YG, ZP vs. YC, and YC vs. YG) were intersected. The Venn diagram showed a total of 24 common KEGG pathways (**Supplementary Figure 4**). Subsequently, a bar chart (**Figure 7**) was used to display these shared differential KEGG pathways, including “pyruvate metabolism”, “isoquinoline alkaloid biosynthesis”, “zein biosynthesis”, “galactose metabolism”, and “cyanate metabolism”. Among them, “metabolic pathways” and “biosynthesis of secondary metabolites” contained the largest number of differential genes. “Cofactor biosynthesis” (across ZP vs. YG, ZP vs. YC, and YC vs. YG) and “amino acid biosynthesis” (across ZP vs. YG and ZP vs. YC) were the next most abundant pathways, indicating that the core regulatory effects of *G. scabra* in the three habitats are concentrated on substance synthesis and metabolism. In the analysis of common KEGG pathways, a total of 50, 33, and 26 DMs related to the biosynthesis of secondary metabolites were identified in the three comparison groups, respectively. To enhance the reliability of results and address the multiple testing issue, the P-values of all enrichment analyses were corrected using the false discovery rate (FDR) method with a correction criterion of FDR < 0.05. In comparison with simple pairwise gene-metabolite correlation analysis, this pathway-level integrated enrichment analysis provides a more systematic and reliable interpretation.

### 3.6 Exploratory correlation network between DEGs and DAMs

**Figure 8:**
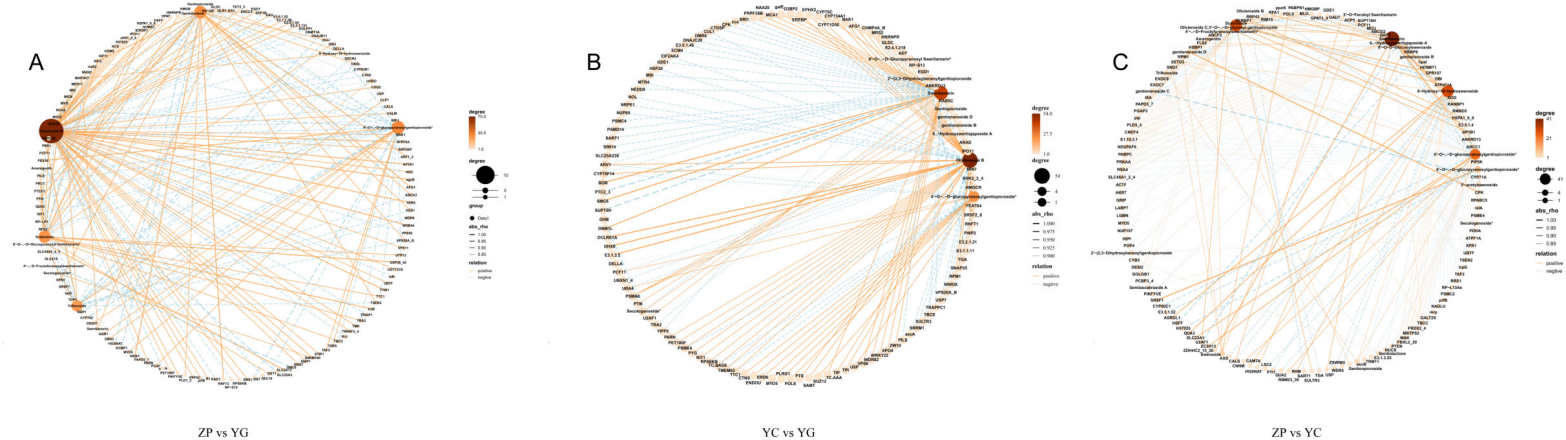
Correlation network analysis of DEGs and DMs in *G. scabra* across three comparison groups. **(A)** Correlation network for the ZP vs. YG comparison group; **(B)** Correlation network for the YC vs. YG comparison group; **(C)** Correlation network for the ZP vs. YC comparison group. Nodes represent DEGs and DMs, with node size proportional to the degree of connectivity (hub nodes are larger). Orange solid lines indicate significant positive correlations, and light blue dashed lines indicate significant negative correlations. Correlation analysis was performed using Spearman’s rank correlation, with all P-values corrected for multiple testing via the false discovery rate (FDR) method. Significant correlations were defined as |ρ| > 0.80 and FDR < 0.05. YG: Wild shrubby *G. scabra*; YC: Wild meadow *G. scabra*; ZP: Cultivated farmland *G. scabra*; FDR, false discovery rate; ρ, Spearman’s correlation coefficient.

Based on numerous studies indicating metabolites related to iridoid biosynthesis (Zhang et al., 2024), 28 target metabolites potentially associated with the quality formation of *G. scabra* medicinal materials were screened (**Supplementary Table 8**). To mitigate false positives arising from integrating count-based (RNA-seq) and intensity-based (metabolomics) data, Spearman’s rank correlation was used, and all p-values were subjected to FDR correction. Significant correlations were defined as |p| > 0.80 with FDR < 0.05. A network was constructed using these stringent criteria (**Figure 8**). Correlation analysis was performed between DEGs and DMs of *G. scabra* from the three habitats. DEGs and DMs with p < 0.05 were selected to construct a correlation network diagram. The rBased on the terpenoid backbone biosynthesis reference pathway in the KEGG database (map00900; https://www.kegg.jp/pathway/map00900), we mapped the key enzymes involved in the biosynthesis of gentiopicroside precursors. This pathway provides a framework for understanding how the differentially expressed genes (e.g., *HMGR, MVD, IDI*) identified in this study participate in gentiopicroside biosynthesis.

Results showed that in the ZP vs YG group, twenty-eight genes were correlated with scabraside content. Among them, *crtISO, EEF1B, malQ, NUG2, PEX16*, and *QUA2* showed a positive correlation with scabraside content in the wild shrubby habitat compared to the cultivated farmland habitat. Twenty-five genes were associated with trifloroside content, and the expression levels of *CLP1, crtISO, MVD, PBS1, SLC44A2_4_5*, and *SRSF7* were positively correlated with trifloroside content. Thirty-one genes were correlated with 4’-O-β-D-glucopyranosylgentiopicroside content. The expression levels of *DDX47, DELLA, GAR1, GBA2, MYO5, PCAF, PEX16, TAF2*, and *TARS* were positively correlated with the content of this metabolite. Thirty-one genes were associated with gentiopicroside content. Among them, *crtISO, DELLA, EEF1B, KCMF1, NUG2, PEX16, QUA2, and SLC44A2_4_5* showed a positive correlation with gentiopicroside content in the wild shrubby habitat compared to the cultivated farmland habitat. Seventy genes were correlated with olivieroside B content. In the YC vs YG group, the DMs associated with a relatively large number of DEGs mainly included olivieroside B, swertiamarin, and 4’-O-β-D-glucopyranosylgentiopicroside. In the ZP vs. YC group, the DMs associated with a relatively large number of DEGs mainly included scabraside, swertiamarin, 8-Hydroxy-10-hydrosweroside, 4’-O-β-D-glucopyranosylgentiopicroside, and 6’-O-β-D-glucopyranosylgentiopicroside. This analysis revealed that several pivotal iridoid glycosides, including gentiopicroside and scabraside, exhibited extensive correlations with gene sets in the ZP vs. YG comparison, which suggests a coordinated regulatory pattern in wild shrub habitats. In contrast, the association patterns diverged in other comparison groups, thereby highlighting the habitat-specific gene-metabolite crosstalk.

This correlation network serves as a hypothesis-generating exploratory analysis, whereas the robust, systems-level insights are obtained from the pathway-based integrative analysis in Section 3.5, and the present analysis further identifies specific candidate gene-metabolite associations to facilitate subsequent functional validation.

## 4 Discussion

In this study, transcriptomic and metabolomic analyses were performed on the roots of *G. scabra* from three habitats (wild shrubby, wild meadow, and cultivated farmland) to investigate the quality differences of *G. scabra* and their underlying regulatory mechanisms.

### 4.1 Habitat-associated metabolic differences in *G. scabra*

Metabolite comparison of *G. scabra* across three habitats revealed that the number of DMs between cultivated farmland and wild shrubby populations was the highest. Enrichment analysis based on the KEGG database indicated that these DMs were predominantly concentrated in pathways related to “metabolic pathways” and “environmental information processing”, which is consistent with the findings of previous studies focusing on the effects of abiotic stress on other plant species (Guo et al., 2020; Zhang et al., 2021; Pan et al., 2025). The “habitat type” in this study represents a comprehensive manifestation of multiple interrelated environmental factors (e.g., light conditions, soil properties, biotic interactions, and anthropogenic management practices). Plants are exposed to both abiotic and biotic stresses in natural environments (Wang et al., 2024; Liang et al., 2024; Yu et al., 2024; Zhang et al., 2024; Wu et al., 2024). Therefore, the observed multi-omics variations in this study should be interpreted as the holistic response of *G. scabra* to the unique composite environmental conditions of different habitats. This observation may be attributed to the distinct environmental conditions between the two habitats: compared with *G. scabra* in wild shrubby habitats, those grown in cultivated farmland are subjected to a simplified vegetation composition and community structure, with no shading from tall vegetation, resulting in sufficient and uniform surface illumination and minimal interference from surrounding vegetation during growth. Additionally, farmland habitats involve artificial tillage and fertilization practices, leading to controllable but easily imbalanced nutrient contents and a high risk of soil compaction due to repeated cultivation. In contrast, wild shrubby habitats are dominated by natural shrubs accompanied by a small number of herbaceous plants, characterized by high species richness, obvious vertical community stratification, high light spot heterogeneity with distinct shaded areas, no artificial disturbance, deep accumulation of organic matter, and a more stable soil structure. These distinct habitat characteristics are associated with the largest number of DMs observed between cultivated farmland and wild shrubby *G. scabra* populations. Notably, the identified DMs were mainly terpenoids, which are the major bioactive components of *G. scabra*. Among these, iridoids represent the core bioactive terpenoids, and the currently isolated iridoids are primarily classified into three categories: ordinary iridoids, 4-noriridoids, and secoiridoids. Specifically, ordinary iridoids include loganic acid, 6’-O-β-D-glucopyranosyl loganic acid, and loganin; 4-noriridoids comprise scrophularoside A, rehmannioside B, and rehmannioside C; and secoiridoids consist of gentiopicroside, sweroside, and swertiamarin (Ye et al., 2023). These iridoid compounds have been reported to exhibit multiple pharmacological activities, including anti-inflammatory, analgesic, hepatoprotective, and antioxidant effects.

Comparison of DMs of *G. scabra* across three habitats revealed that the DMs highly expressed in cultivated farmland-grown *G. scabra* were predominantly amino acids, their derivatives, and phenolic acids. This phenomenon may be attributed to the sufficient nitrogen sources provided by artificial fertilization (high nitrogen and phosphorus levels) in farmlands, which facilitate amino acid synthesis (Arnold et al., 2015). As core building blocks of proteins and enzymes, the high expression of amino acids enables rapid cell structure construction and accelerated biomass accumulation (Dorward et al., 2019). Meanwhile, farmland habitats lack extreme drought and intense interspecific competition, and artificial weeding and pest control replace the need for plants to activate their own defense mechanisms. Consequently, carbon and nitrogen resources are preferentially allocated to growth-related metabolites such as amino acids and phenolic acids. Phenolic acids possess weak antibacterial activity (Kalinowska et al., 2024; Li et al., 2015), allowing the maintenance of basic defense without substantial energy consumption. Among the DMs highly expressed in wild meadow-grown *G. scabra*, a large proportion belong to lignans and coumarins, which may be associated with stress factors in meadow habitats, including local drought, uneven soil nutrient distribution, and interspecific competition. High lignan expression enhances cell wall rigidity (Shad et al., 2024), which not only supports the elongation of herbaceous stems to compete for light and resist wind, rain, and lodging but also reduces herbivorous insect feeding and pathogen infection while decreasing transpiration-induced water loss, adapting to the fluctuating water conditions caused by occasional drought in meadows. Additionally, the high expression of lignans and coumarins can act as allelochemicals to inhibit the growth of surrounding weeds (Wang and Chang, 2019), reducing nutrient competition. These compounds also regulate osmotic balance and enhance antioxidant capacity to mitigate drought stress damage, conferring both stress tolerance and competitive advantages to *G. scabra* in meadow habitats.

Integrated metabolomic and transcriptomic analyses (**Figures 1**, **8**) identified habitat-specific metabolic signatures and regulatory genes in Gentiana scabra. PCA (**Figure 1A**, PC1 = 54.54%) clearly separated the three habitat groups, confirming habitat as the dominant driver of metabolic variation. Correlation network analysis (**Figure 8**) revealed that quality-marker iridoid glycosides (gentiopicroside, scabraside) are co-regulated with key DEGs (*crtISO, DELLA, MVD*), demonstrating coordinated transcriptional control of bioactive compounds. This multi-omics approach linked phenotype, metabolism and genotype, providing robust candidate genes for *G. scabra* molecular breeding.

### 4.2 Regulation of terpenoid biosynthesis pathways

Based on the terpenoid backbone biosynthesis reference pathway in the KEGG database (map00900; https://www.kegg.jp/pathway/map00900), we mapped the key enzymes involved in the biosynthesis of gentiopicroside precursors. This pathway provides a framework for understanding how the DEGs (e.g., HMGR, MVD, IDI) identified in this study participate in gentiopicroside biosynthesis. The main bioactive component of *G. scabra* is gentiopicroside, and its biosynthetic pathway consists of two stages: the generation of the precursor isopentenyl diphosphate (*IPP*) and the formation of secoiridoids. Notably, there are two distinct biosynthetic pathways for IPP, a key precursor of terpenoids (Dai et al., 2023): the mevalonate (MVA) pathway in the cytoplasm and the methylerythritol phosphate (MEP) pathway in plastids. Among these pathways, two key enzymes involved in the MVA pathway—diphosphomevalonate decarboxylase (*MVD*) and 3-hydroxy-3-methylglutaryl-CoA reductase (*HMGR*)—exhibited significant differences in *G. scabra* across the three habitats. Additionally, isopentenyl diphosphate delta-isomerase (*IDI*), a key enzyme in the MEP pathway, showed significant variations, which collectively affect the biosynthesis of terpenoids. Mevalonate diphosphate decarboxylase (*MVD*) is an ATP-dependent enzyme that catalyzes the phosphorylation and decarboxylation of (R)-mevalonate 5-diphosphate to form isopentenyl pyrophosphate (*IPP*) in the mevalonate (MVA) pathway (Rossoni et al., 2015). Relevant studies have shown that 3-hydroxy-3-methylglutaryl-CoA reductase (*HMGR*), a key enzyme involved in terpenoid biosynthesis in *Santalum album* L. is one of the rate-limiting enzymes essential for the synthesis of sandalwood sesquiterpenes (Niu et al., 2025). Relevant studies have demonstrated that the overexpression of GbERF4 in Nicotiana tabacum (tobacco) significantly increased terpenoid content and upregulated the expression of key enzyme genes in the tobacco terpenoid biosynthesis pathway, including 3-hydroxy-3-methylglutaryl-CoA reductase (*HMGR*) (Zheng et al., 2024). Isopentenyl diphosphate isomerase (*IDI*) catalyzes the interconversion between isopentenyl diphosphate (*IPP*) and dimethylallyl diphosphate (*DMAPP*) (Pankratov et al., 2016; Wu et al., 2005; Zheng et al., 2025; Chunmei et al., 2025). This reaction is an essential step for mevalonate to enter the isoprenoid biosynthetic pathway, as IDI-mediated interconversion of IPP and DMAPP provides the key precursors required for terpenoid biosynthesis.

### 4.3 Selection and functional significance of qRT-PCR validated genes

The six genes subjected to qRT-PCR validation were selected based on their established and critical roles in the terpenoid backbone biosynthesis and iridoid glycoside formation pathways (Zhang et al., 2024). *HMGR*, *MVD*, and *IDI* encode core enzymes in the mevalonate (MVA) and methylerythritol phosphate (MEP) pathways, which supply the universal isoprenoid precursors *IPP* and *DMAPP* required for the biosynthesis of all terpenoid metabolites (Rohmer, 1999; Tholl, 2006). *DELLA* proteins function as key negative regulators in gibberellin signaling, governing the metabolic trade−off between plant growth and secondary metabolism (Hauvermale et al., 2012). The gene *crtISO* (carotenoid isomerase) participates in carotenoid biosynthesis, a pathway that shares common upstream precursors with the iridoid pathway and thus serves as an indicator of metabolic flux partitioning (Isaacson et al., 2002). *PILS* (PIN−LIKE) auxin efflux carriers modulate plant growth and stress adaptation, which indirectly influence the accumulation of secondary metabolites (Barbez et al., 2012). The highly consistent expression profiles between RNA−seq and qRT−PCR for these functionally interconnected genes confirm the reliability of our transcriptomic data and support the coordinated regulation of primary and secondary metabolism in *G. scabra* across distinct habitat conditions.

### 4.4 Identification of habitat-responsive key genes

Plants regulate genes within their bodies to adapt to different living environments, resulting in DEGs. The expression levels of four genes, namely *HSFF, SCPL-I, LOC107801770*, and *CYP71D55*, showed significant variations in *G. scabra* across the three habitats, suggesting that these four genes may represent candidate regulators involved in habitat adaptation. Heat shock transcription factors (*HSFs*) are key regulators of plant responses to environmental stresses and growth and development processes. When plants are exposed to abiotic stresses such as high temperature and drought, *HSFs* can recognize and bind to the heat shock elements (*HSEs*) in the promoter regions of downstream genes, thereby activating the transcriptional expression of related genes and regulating plant stress responses (Zhang et al., 2026). HSFs act as a central hub for trade-offs to mediate stress resilience and growth and development in plants (Bakery et al., 2024). Relevant studies have demonstrated that the *HSF* gene family serves as a key regulator of the response mechanism to high-temperature stress in *Paeonia suffruticosa* (Zhang et al., 2025). Studies on homologous genes in wheat have revealed that HSFs enhance the capacity for protein homeostasis maintenance by activating downstream target genes (Wei et al., 2025). The differential expression of *HSFF* (a member of this gene family) in *G. scabra* across different habitats indicates the presence of a perception mechanism for abiotic stresses such as high temperature and drought in this species. *SCPL-I* (Serine Carboxypeptidase-Like) proteins belong to the serine carboxypeptidase protein family. The *SCPL* acyltransferase family is localized in the vacuole (Liu et al., 2024). These enzymes utilize acyl-CoA as acyl donors to transfer acyl groups to specific plant metabolites, thereby catalyzing acylation modifications (Cao et al., 2024), and play a crucial role in the diversification of plant metabolites (Yao et al., 2022). In *Carya cathayensis*, the expression level of *CcSCPL4* from the *SCPL* gene family exhibited significant changes following drought, low-temperature and salt stress treatments, with the highest expression level detected under drought stress, which consequently enhances the drought resistance of plants. This finding is consistent with the results of our previous study that drought exerts a significant effect on the quality of *G. scabra*, indicating that the expression changes of this gene may affect the acylation modifications of phenolic acids, iridoid glycosides and other metabolites in *G. scabra*, thereby altering their stability and biological activity.*CYP71D55* belongs to the *CYP71D* subfamily within the cytochrome P450 (*CYP450*) superfamily. Extensive studies have demonstrated that the *CYP71D* subfamily acts as a key driver of plant secondary metabolic diversification, which specifically catalyzes oxidative reactions such as hydroxylation and epoxidation to generate structurally diverse end products. For instance, in Gentianales, a monophyletic clade (the GAS clade) within the *CYP71D* subfamily has driven the radiative evolution of thousands of monoterpenoid indole alkaloid structures through gene duplication and neofunctionalization (Besseau et al., 2025). In *Salvia miltiorrhiza*, members of the *CYP71D* subfamily are responsible for catalyzing the formation of the characteristic furan D-ring of tanshinones, which constitutes the key structural moiety underlying their pharmacological activities (Li S. et al., 2022). Studies on *Hedyotis diffusa* have indicated that *CYP71D55* is involved in the potential post-modification processes of the iridoid biosynthetic pathway and acts as a key contributor to this metabolic process (Chen et al., 2025). Based on these precedents, we hypothesize that *CYP71D55* in the present study is most likely responsible for catalyzing specific oxidative modification steps (e.g., hydroxylation) during the late stage of iridoid (e.g., gentiopicroside) biosynthesis in *G. scabra.* Its differential expression across different habitats directly leads to variations in the final metabolites, thus contributing to the formation of habitat specificity. This finding provides a definitive molecular mechanistic explanation for the classic question in Chinese materia medica regarding why the same plant species produces distinct chemical constituents in different environments. *LOC107801770* is a gene with no definitive functional annotation to date. Through co-expression network analysis, we found that it was highly co-expressed with multiple well-characterized genes in the iridoid biosynthetic pathway. Combined with the characteristics of its putative oxidoreductase domain, we predict that this gene may encode a novel enzyme involved in the scaffold construction or modification of iridoids. In summary, we propose an integrated model to explain the molecular mechanism by which habitats shape the quality of *G. scabra* medicinal materials: *HSFF* acts as an upstream stress sensor and transcriptional regulator to initiate global metabolic reprogramming; *CYP71D55* serves as a core oxidase responsible for establishing the structural diversity of iridoid scaffolds; *LOC107801770*, as a putative novel member, may be involved in specific branch pathways; and *SCPL-I* mediates the fine acylation modification of precursors in the downstream pathway, ultimately leading to the formation of a complete and habitat-specific metabolite profile.

Plant secondary metabolism is a product of the long-term evolutionary interaction between plants and their environment. Through correlation analysis of DEGs and key DMs in this study, it was found that “metabolic pathways” and “biosynthesis of secondary metabolites” contained the largest number of DEGs, followed by “cofactor biosynthesis” and “amino acid biosynthesis” with the next highest abundance of differential genes. This indicates that the core regulatory effects of *G. scabra* across the three habitats are concentrated on substance synthesis and metabolism. Among these pathways, “cofactor biosynthesis” and “amino acid biosynthesis” belong to primary metabolism, which together with secondary metabolism form the overall framework for substance synthesis and transformation. Meanwhile, amino acid biosynthesis provides core precursors for secondary metabolites, and cofactors serve as essential auxiliary substances for metabolic enzyme activity, collectively ensuring the efficient operation of secondary metabolism. Numerous studies have demonstrated that environmental factors (such as light intensity, temperature, and drought stress) can affect plant photosynthesis, protective enzyme systems, and secondary metabolite biosynthesis pathways (Imran et al., 2021; Rudy, 2014; Li W. N. et al., 2022; Liu et al., 2025). For example, Light intensity affects the biosynthesis of amino acids and their coenzymatic factors in *Gentiana macrophylla* Pall., thereby further regulating the synthesis of secondary metabolites such as loganic acid (Fu et al., 2024). Studies on *G. scabra* have demonstrated that the soil microenvironment coordinately modulates the synthesis of secondary metabolites, which in turn influences the gentiopicroside content (Hou et al., 2025). Under low-temperature stress, plants can enhance acetyl-CoA supply by strengthening primary metabolism and allocate carbon sources to terpenoid compounds through coordinating the activation of the MVA pathway and inhibition of the MEP pathway (Zheng et al, 2025). Based on this, it is speculated that under different habitats, changes in cofactors of *G. scabra* may alter amino acid biosynthesis processes, regulate primary metabolic pathways, and ultimately affect secondary metabolite biosynthesis, thereby influencing the quality formation of *G. scabra*.

### 4.5 Considerations for future research

While this study provides integrated insights into habitat-associated metabolic and transcriptomic variation in *G. scabra*, several aspects warrant further investigation. First, although three biological replicates per group meet the minimum requirement for differential expression analysis using DESeq2 with FDR correction, the statistical power for complex network-based inference is inherently limited. Therefore, the gene–metabolite correlations identified here should be interpreted as exploratory, and the candidate genes proposed would benefit from targeted functional validation in future studies. Second, the sampling strategy employed across a broad geographic range was designed to capture the natural ecological diversity of *G. scabra*. Nevertheless, the observed differences may reflect a combination of habitat conditions and underlying population genetic variation. Future studies employing common garden experiments with genetically uniform materials will be valuable to further disentangle these factors. Third, while we have incorporated environmental descriptors and soil nutrient data to support our interpretations, these measurements remain correlative. Controlled experiments with manipulated environmental factors will be necessary to establish causal relationships between specific habitat conditions and the observed omics patterns. Collectively, these considerations highlight promising avenues for future research to build upon the findings presented here.

## 5 Conclusion

In this study, metabolomic and transcriptomic analyses were conducted on *G. scabra* from wild shrubby, wild meadow, and cultivated farmland habitats. The key findings are as follows: DMs of *G. scabra* across the three habitats were mainly enriched in “metabolic pathways”; *G. scabra* from cultivated farmland showed higher similarity to that from wild meadow, while exhibiting relatively greater differences from wild shrubby *G. scabra*. The expression levels of four genes—*HSFF, SCPL-I, LOC107801770*, and *CYP71D55*—can serve as candidate markers for distinguishing *G. scabra* among the three habitats. Integrated analysis of their differential expression patterns and functional annotations suggests a coordinated regulatory network in response to habitat-specific conditions: HSFF likely acts as an upstream stress-responsive transcription factor, initiating global metabolic reprogramming; *CYP71D55* is proposed to catalyze key oxidative modifications in iridoid biosynthesis, contributing to the structural diversification of bioactive compounds; *SCPL-I* may mediate downstream acylation of secondary metabolites, influencing their stability and bioactivity; and *LOC107801770*, though uncharacterized, is predicted to encode a novel enzyme involved in iridoid scaffold modification based on co-expression and domain analyses. Collectively, these findings provide a mechanistic framework for understanding how distinct habitats shape the quality of *G. scabra* through the regulation of specific gene modules. The high-quality expression of *G. scabra* may be associated with the regulation of *HMGR, MVD, IDI*, and other key genes in the terpenoid backbone biosynthesis pathway. Further analysis of the regulatory relationships between ecological factors, cultivation interventions, and their corresponding target genes will lay a foundation for optimizing *G. scabra* germplasm resources and constructing advanced ecological cultivation models.

## Data Availability

Raw transcriptomic sequence data (GSA: CRA038965; https://ngdc.cncb.ac.cn/gsa) and mass spectrometry-derived metabolomic data (OMIX: OMIX015217; https://share.cncb.ac.cn/IBgeXWua2T/OMIX015217/) reported in this study have been deposited in the Genome Sequence Archive (GSA) and Open Archive for Miscellaneous Data (OMIX), respectively, at the National Genomics Data Center (NGDC), China National Center for Bioinformation/Beijing Institute of Genomics, Chinese Academy of Sciences.
