# Supplementary material for: Metabolome and transcriptome analyses reveal metabolic differences and identify candidate regulatory factors among three habitat types of Gentiana scabra Bunge
Source: Front Plant Sci. 2026 Apr 29;17:1761588. doi: 10.3389/fpls.2026.1761588 (PMC13167545; doi:10.3389/fpls.2026.1761588)
Supplement: Supplementary file 1 [file Supplementaryfile1.docx]

# Supplementary Material

Table S1. Statistics of assembly results

| Type | Number | Mean Length | N50 | N90 |
| --- | --- | --- | --- | --- |
| Transcript | 162069 | 1151 | 1890 | 482 |
| Unigene | 93076 | 1414 | 2076 | 639 |

Table S2. Summary of sequencing alignment metrics.

| Sample | Total Read pairs | Total mapped reads | Uniq mapped reads | Multiple mapped reads |
| --- | --- | --- | --- | --- |
| YG-1 | 21626875 | 18,327,752(84.75%) | 5,424,090(25.08%) | 12,903,662(59.66%) |
| YG-2 | 20187717 | 17,116,637(84.79%) | 5,000,672(24.77%) | 12,115,965(60.02%) |
| YG-3 | 24883448 | 21,564,393(86.66%) | 6,360,576(25.56%) | 15,203,817(61.10%) |
| YC-1 | 25480465 | 22,037,739(86.49%) | 6,435,958(25.26%) | 15,601,781(61.23%) |
| YC-2 | 25744841 | 21,774,297(84.58%) | 6,814,851(26.47%) | 14,959,446(58.11%) |
| YC-3 | 23225458 | 20,005,353(86.14%) | 5,949,097(25.61%) | 14,056,256(60.52%) |
| ZP-1 | 26240311 | 22,878,824(87.19%) | 6,751,763(25.73%) | 16,127,061(61.46%) |
| ZP-2 | 23279574 | 20,055,453(86.15%) | 5,700,969(24.49%) | 14,354,484(61.66%) |
| ZP-3 | 24810160 | 21,065,401(84.91%) | 6,392,763(25.77%) | 14,672,638(59.14%) |

Table S3. Primer Sequences

| Gene |  | sequence（5'-3'） | product size |
| --- | --- | --- | --- |
| HMGR | F | CTGAAGGGTGTTTGGTGGCT | 126bp |
|  | R | TGGCGGTGCTAAACCTAACA |  |
| MVD | F | CACCTGGGGAATGCATAAACTG | 100bp |
|  | R | TATCAGAGACTTGTGGGCAGG |  |
| IDI | F | CGCCTCATGTTTGAAGACGAA | 137bp |
|  | R | AGGAACACGCTAAATGCCCTAT |  |
| DELLA | F | GGCTGCAAGTGAACTCCTGA | 106bp |
|  | R | TATGCGTGCTTCAAGACCGT |  |
| crtISO | F | TTGAGGAGTGGGAGGGTCTT | 150bp |
|  | R | TGTGTGTCTTCGGTGTAGCC |  |
| PILS | F | TTCTACCTTGTGGTTCCCCG | 100bp |
|  | R | AGTGCAATAACTGTGCGTGG |  |
| HD-ZIP | F | GCCAAAGGCACCAAACAGAA | 120bp |
|  | R | ATCGAACTGTCTGGGGGTCT |  |
| Actin | F | TGTTGCCCTTGACTACGAGC | 128bp |
|  | R | GGACTTCAGGGCAACGGAAT |  |

Table S4. Highly expressed differential metabolites in different habitats

|  | Metabolite Name | High-expression Group |
| --- | --- | --- |
| 1 | 10-Hydroxyligustroside | ZP |
| 2 | 3,4-Dihydroxy-L-phenylalanine (L-Dopa) |  |
| 3 | 3-Hydroxy-L-phenylalanine |  |
| 4 | 3-hydroxyphenylacetic acid* |  |
| 5 | 4-O-(6'-O-Glucosylcaffeoyl)-4-hydroxybenzoic acid |  |
| 6 | 6-O-methylguanine |  |
| 7 | Caffeic acid |  |
| 8 | codonopilodiynoside F |  |
| 9 | codonopilodiynoside G |  |
| 10 | Dihydrosphingosine |  |
| 11 | Isochlorogenic acid C* |  |
| 12 | kwanzoquinone C |  |
| 13 | L-Homocysteine |  |
| 14 | Ligstroside |  |
| 15 | L-Tyrosine |  |
| 16 | Macrophylloside B |  |
| 17 | Saln005276 |  |
| 18 | Methyl gallate |  |
| 19 | N-(2-Hydroxy-4-methoxyphenyl)acetamide |  |
| 20 | naphthisoxazol A |  |
| 21 | Protocatechualdehyde |  |
| 22 | S-Methyl-L-cysteine |  |
| 23 | 6-O-Vanilloylajugol | YC |
| 24 | Cichoriin |  |
| 25 | Daphnin* |  |
| 26 | davidioside C |  |
| 27 | Dihydromarein* |  |
| 28 | Esculin (6,7-Dihydroxycoumarin-6-O-glucoside)* |  |
| 29 | Gingerglycolipid B |  |
| 30 | Luteolin-7-O-glucuronide |  |
| 31 | Myricetin-3-O-rhamnoside (Myricitrin) |  |
| 32 | Secologanin |  |
| 33 | 7-O-(4''-O-glucosyl)coumaroyl-loganic acid | YG |
| 34 | Arg-Cys-Tyr |  |
| 35 | Hinokitiol Acetate |  |

Asterisks (*) indicate that the metabolites exhibit particularly significant differential expression in the corresponding groups.

Table S5. Summary of raw RNA-seq data of *Gentiana scabra*

| Group | Sample | Raw Reads | Clean Reads | Clean Bases | Error Rate | Q20(%) | Q30(%) | GC Content(%) |
| --- | --- | --- | --- | --- | --- | --- | --- | --- |
| 1 | YG-1 | 44954772 | 43253750 | 6.49 | 0.03 | 97.81 | 93.52 | 43.52 |
| 1 | YG-2 | 41902452 | 40375434 | 6.06 | 0.03 | 97.78 | 93.44 | 43.5 |
| 1 | YG-3 | 51700146 | 49766896 | 7.47 | 0.03 | 97.84 | 93.57 | 43.78 |
| 2 | YC-1 | 52857118 | 50960930 | 7.64 | 0.03 | 97.77 | 93.4 | 43.54 |
| 2 | YC-2 | 53468276 | 51489682 | 7.72 | 0.03 | 97.79 | 93.46 | 43.81 |
| 2 | YC-3 | 48037586 | 46450916 | 6.97 | 0.03 | 97.85 | 93.61 | 43.73 |
| 3 | ZP-1 | 54551386 | 52480622 | 7.87 | 0.03 | 97.79 | 93.47 | 43.88 |
| 3 | ZP-2 | 48441316 | 46559148 | 6.98 | 0.03 | 97.78 | 93.43 | 43.73 |
| 3 | ZP-3 | 51205492 | 49620320 | 7.44 | 0.03 | 97.63 | 93.1 | 43.31 |

Table S6. Gene annotation results of *Gentiana scabra*transcriptome

| Database | Number Unigenes | Percentage (%) |
| --- | --- | --- |
| KEGG | 47143 | 50.65 |
| Nr | 60229 | 64.71 |
| SwissProt | 45653 | 49.05 |
| TrEMBL | 59798 | 64.25 |
| KOG | 37797 | 40.61 |
| GO | 52680 | 56.6 |
| Pfam | 41985 | 45.11 |
| Annotated in at least one Database | 62131 | 66.75 |
| Total Unigenes | 93076 | 100 |

Table S7. KEGG Pathways of Differentially Expressed Genes (DEGs)

|  | Name of KEGG Pathway |
| --- | --- |
| 1 | Spliceosome |
| 2 | Ribosome |
| 3 | N-Glycan biosynthesis |
| 4 | Nucleotide excision repair |
| 5 | RNA degradation |
| 6 | Endocytosis |
| 7 | Mismatch repair |
| 8 | Phagosome |
| 9 | Basal transcription factors |
| 10 | Homologous recombination |
| 11 | Proteasome |
| 12 | Terpenoid backbone biosynthesis |
| 13 | Various types of N-glycan biosynthesis |
| 14 | Ribosome biogenesis in eukaryotes |
| 15 | Peroxisome |
| 16 | Nucleocytoplasmic transport |
| 17 | alpha-Linolenic acid metabolism |
| 18 | Sesquiterpenoid and triterpenoid biosynthesis |
| 19 | DNA replication |
| 20 | Protein processing in endoplasmic reticulum |
| 21 | Fatty acid degradation |
| 22 | Isoflavonoid biosynthesis |
| 23 | MAPK signaling pathway - plant |
| 24 | mRNA surveillance pathway |
| 25 | Plant-pathogen interaction |
| 26 | Fatty acid elongation |
| 27 | RNA polymerase |

Table S8. 28 Candidate Metabolites Associated with Quality Formation of *Gentiana scabra* Medicinal Materials

|  | Compounds | Formula |
| --- | --- | --- |
| 1 | 6β-Hydroxyswertiajaposide A | C_17_H_24_O_11_ |
| 2 | 8-Hydroxy-10-hydrosweroside | C_16_H_24_O_10_ |
| 3 | Gentiopicroside | C_16_H_20_O_9_ |
| 4 | Olivieroside C;3'-O-β-D-glucosyl gentiopicroside | C_22_H_30_O_14_ |
| 5 | 6'-O-D-Glucosylsweroside | C_22_H_32_O_14_ |
| 6 | Scabraside | C_40_H_44_O_20_ |
| 7 | Gentiolactone | C_10_H_12_O_5_ |
| 8 | 3'-acetylsweroside | C_18_H_24_O_10_ |
| 9 | gentiananoside C | C_18_H_26_O_11_ |
| 10 | gentiananoside D | C_18_H_26_O_11_ |
| 11 | 4'-β-D-Fructofuranosylswertiamarin* | C_22_H_32_O_15_ |
| 12 | Secologanoside* | C_16_H_22_O_11_ |
| 13 | Olivieroside B | C_25_H_28_O_11_ |
| 14 | Amarogentin | C_29_H_30_O_13_ |
| 15 | 8-Epiloganic acid | C_16_H_24_O_10_ |
| 16 | gentiananoside B | C_16_H_20_O_9_ |
| 17 | 3'-O-Feruloyl Swertiamarin | C_26_H_30_O_13_ |
| 18 | Loganin* | C_17_H_26_O_10_ |
| 19 | 6'-O-β-D-glucopyranosylgentiopicroside* | C_22_H_30_O_14_ |
| 20 | 4'-O-β-D-glucopyranosylgentiopicroside* | C_22_H_30_O_14_ |
| 21 | 2'-(2,3-Dihydroxybenzoyl)gentiopicroside | C_23_H_24_O_12_ |
| 22 | Sweroside | C_16_H_22_O_9_ |
| 23 | Trifloroside | C_35_H_42_O_20_ |
| 24 | 6'-O-β-D-Glucopyranosyl Swertiamarin* | C_22_H_32_O_15_ |
| 25 | Gentiascabraside A | C_17_H_24_O_11_ |
| 26 | angustiamarin | C_26_H_30_O_13_ |
| 27 | 2'-(o,m-Dihydroxybenzyl) sweroside | C_23_H_26_O_12_ |
| 28 | Swertiamarin | C_16_H_22_O_10_ |

Table S9. DEGs Numbers and Alterations Before and After FDR Correction Across Comparison Groups

| Comparison Group | Number of DEGs Screened by Raw P-value | Number of DEGs After FDR Correction | Number of DEGs Removed | Key Retained DEGs (Examples) | Impact on Pathway Enrichment | Notes |
| --- | --- | --- | --- | --- | --- | --- |
| ZP vs YG | 1283 | 917 | 366 | HSFF; SCPL-I; LOC107801770; CYP71D55 | Yes (Attenuation of partial pathways) | Retained core DEGs still support the main conclusions |
| ZP vs YC | 839 | 605 | 234 | LOC107801770; HSFF | No | Most removed DEGs show low expression or low fold change |
| YC vs YG | 878 | 645 | 233 | HSFF、SCPL-I | Yes (Reduction of partial enriched terms) | Core marker genes remain statistically significant |

Table S10. DMs Numbers and Alterations Before and After FDR Correction Across Comparison Groups

| Comparison Group | Number of DMs Screened by Raw P-value | Number of DMs After FDR Correction | Number of DMs Removed | Key Retained DMs (Examples) | Impact on Pathway Enrichment | Notes |
| --- | --- | --- | --- | --- | --- | --- |
| ZP vs YG | 642 | 510 | 132 | genipin-1-O-gentiobioside;  secologanin;  4'-O-β-D-glucopyranosylgentiopicroside; | Yes (Reduction of partial enriched terms) | Retained DMs still include the core metabolites of interest |
| ZP vs YC | 458 | 348 | 110 | apigenin-5-O-glucoside;  secologanin; | Yes (Reduction of partial enriched terms) | Retained DMs still include the core metabolites of interest |
| YC vs YG | 454 | 309 | 145 | homogentisic acid;  scabraside | Yes (Reduction of partial enriched terms) | Retained DMs still include the core metabolites of interest |

Table S11. Environmental characteristics of the sampling sites

| **Sample Code** | **Habitat Type** | **Canopy Cover(%)** | **Soil Type** | **Soil pH** | **Organic Matter (g/kg)** | **Total N (g/kg)** | **Available P (mg/kg)** | **Available K (mg/kg)** | **Slope (°)** |
| --- | --- | --- | --- | --- | --- | --- | --- | --- | --- |
| A1-1 | Wild shrubland | 32 | Brown soil | 6.7 | 28.3 | 1.82 | 12.2 | 98.3 | 14.4 |
| A1-2 | Wild shrubland | 37 | Brown soil | 6.9 | 30.2 | 2.04 | 13.3 | 102.5 | 13.7 |
| A1-3 | Wild shrubland | 35 | Brown soil | 6.8 | 27.7 | 1.91 | 11.8 | 95.9 | 17.2 |
| B2-1 | Wild meadow | 9 | Meadow siol | 7.3 | 35.4 | 2.38 | 15.3 | 112.3 | 2.1 |
| B2-2 | Wild meadow | 8 | Meadow siol | 7.2 | 36.5 | 2.51 | 15.9 | 116.8 | 4.3 |
| B2-3 | Wild meadow | 6 | Meadow siol | 7.1 | 34.9 | 2.42 | 16.1 | 110.9 | 3.6 |
| C3-1 | Cultivated farmland | 0 | Brown soil | 6.5 | 22.5 | 1.66 | 28.5 | 156.5 | 0.5 |
| C3-2 | Cultivated farmland | 0 | Brown soil | 6.4 | 21.7 | 1.57 | 27.9 | 152.1 | 1.4 |
| C3-3 | Cultivated farmland | 0 | Brown soil | 6.6 | 23.4 | 1.71 | 29.2 | 160.4 | 0.8 |


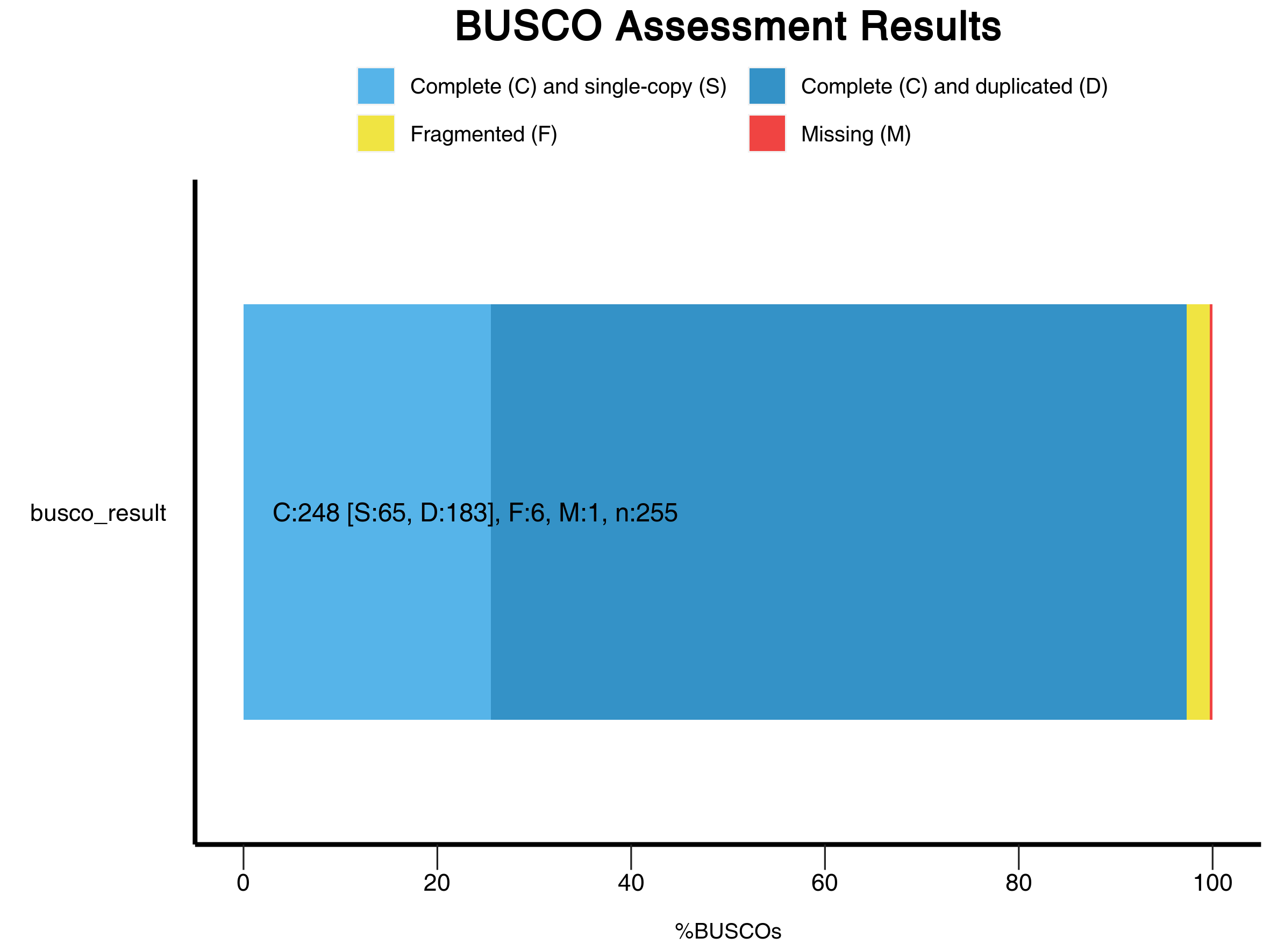


Fig. S1. BUSCO assessment results


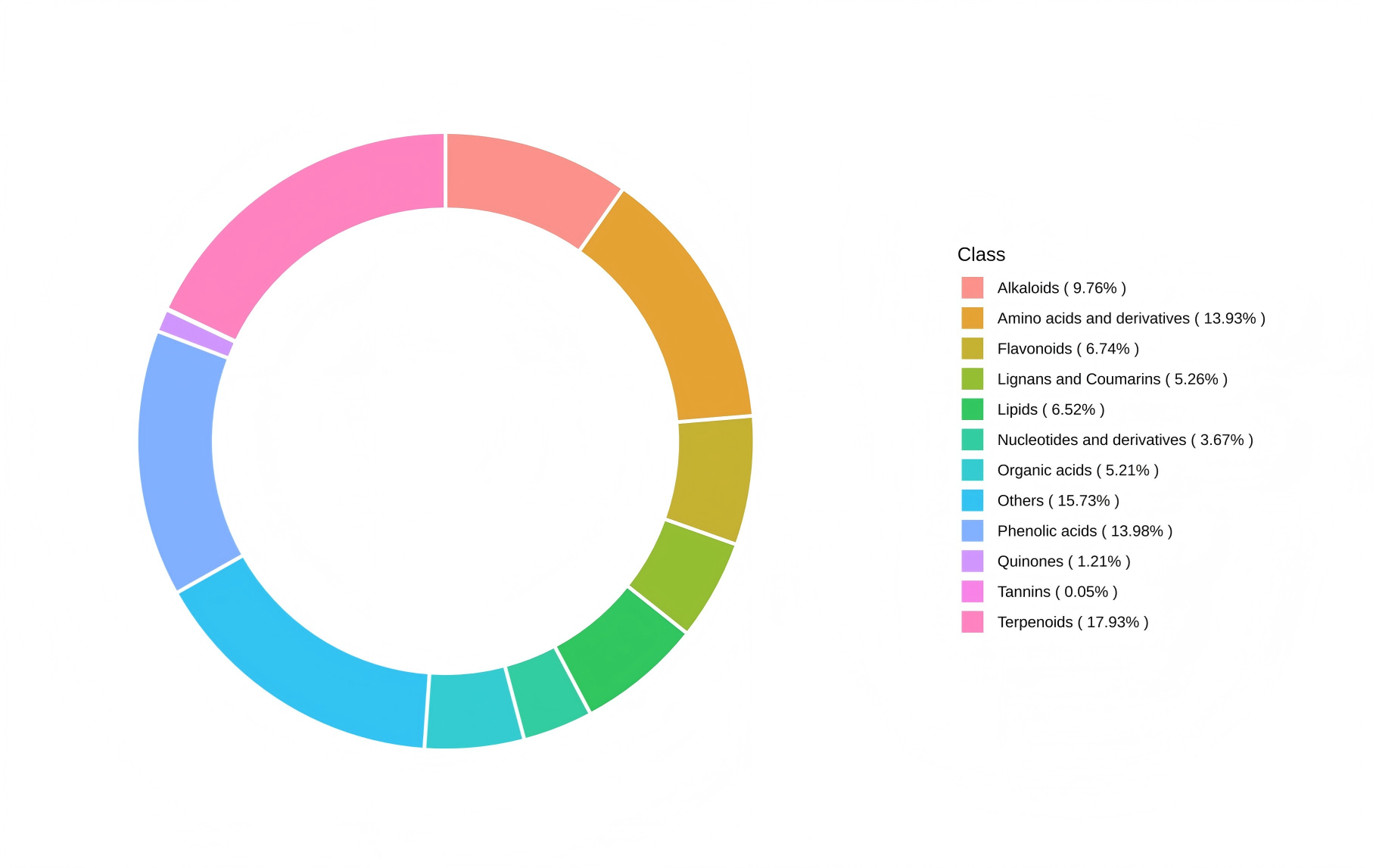


Figure S2. Circular Diagram of Metabolite Category Composition


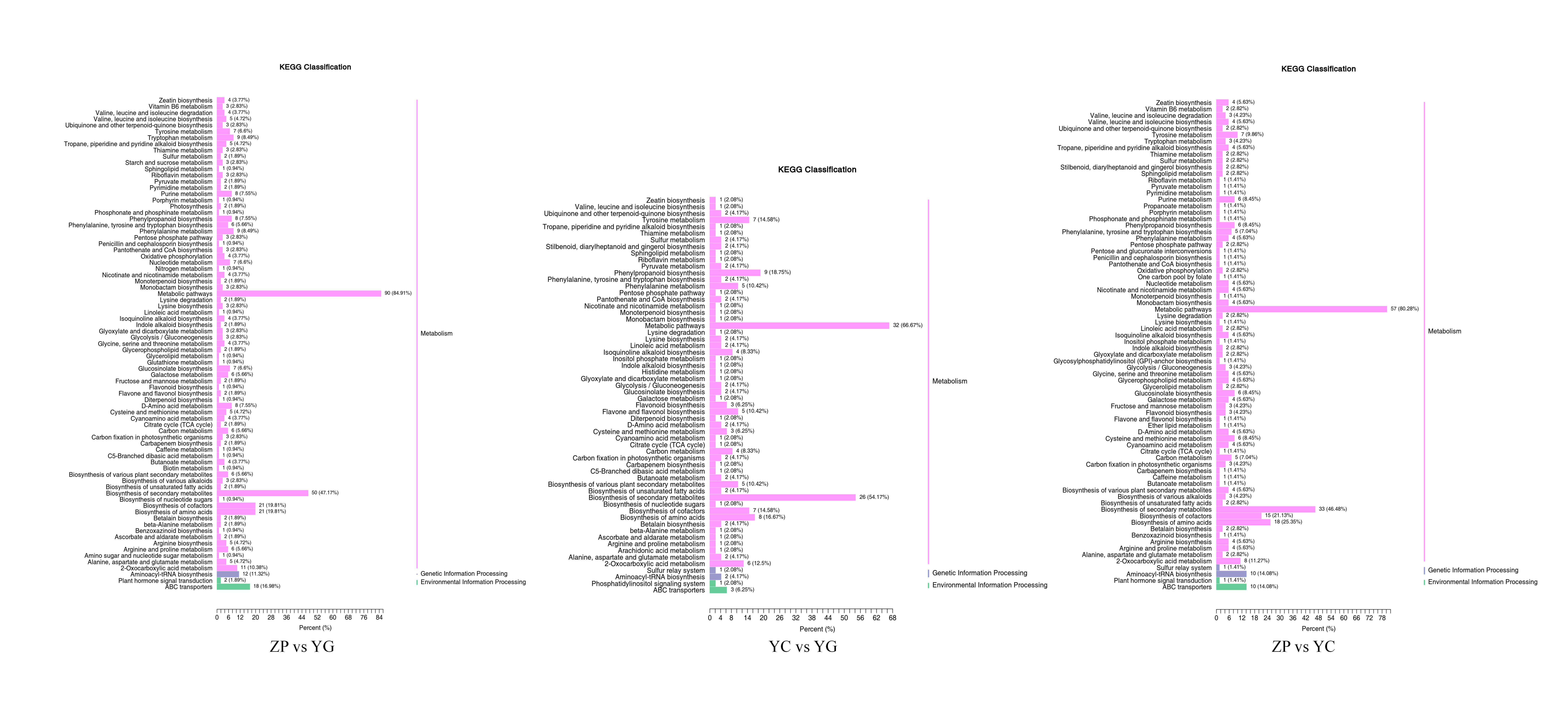


Figure S3. KEGG Enrichment Analysis of Differential Metabolites (DMs) Among Different Comparison Groups


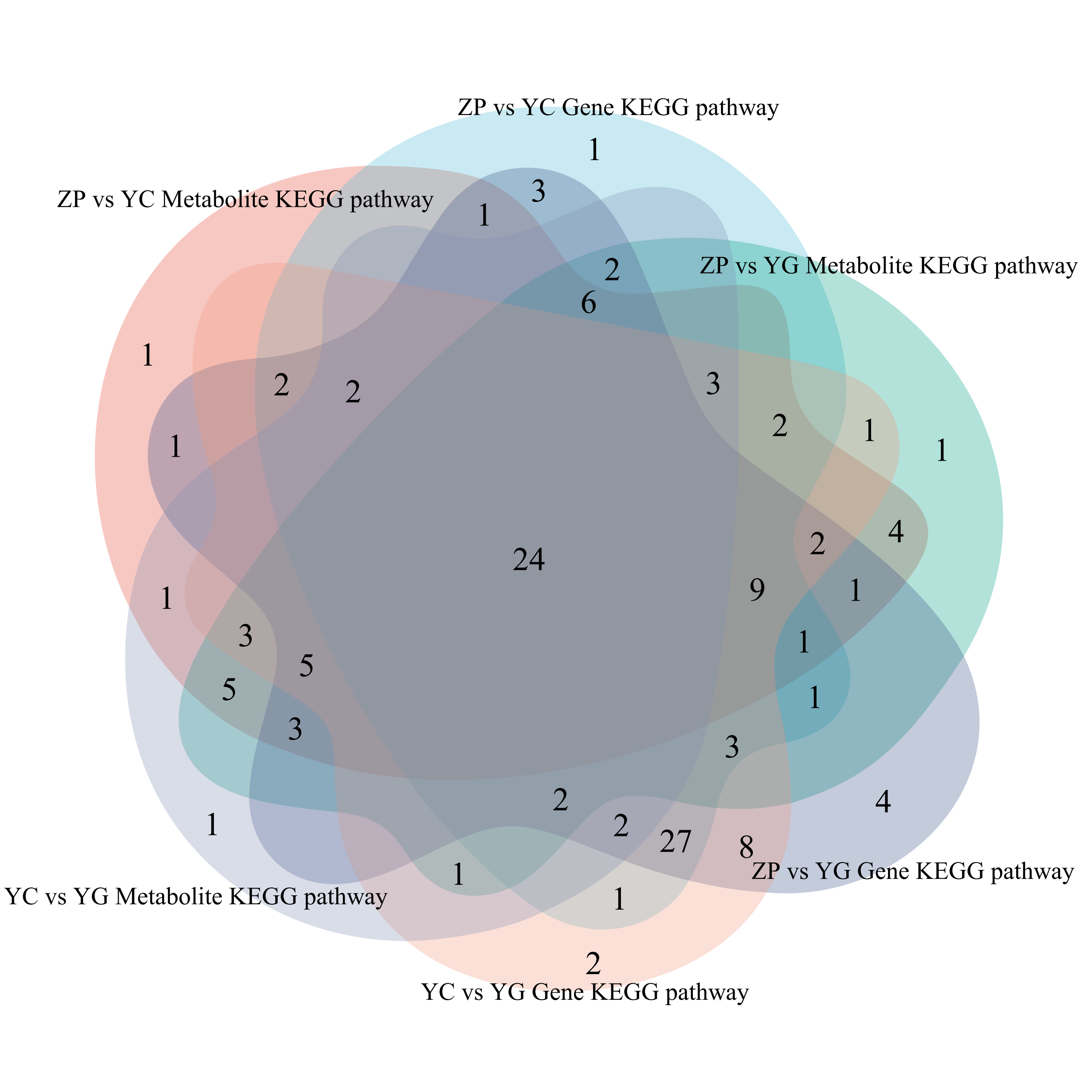


Figure S4. Venn Diagrams of KEGG Pathways for Differentially Expressed Genes (DEGs) and Differential Metabolites (DMs) Among *Gentiana scabra* from Different Habitats
